# Analyzing the Diversity of MYB Family Response Strategies to Drought Stress in Different Flax Varieties Based on Transcriptome Data

**DOI:** 10.3390/plants13050710

**Published:** 2024-03-02

**Authors:** Fan Zhang, Ying Liu, Jie Ma, Shaofeng Su, Liyu Chen, Yuchen Cheng, Siqin Buter, Xiaoqing Zhao, Liuxi Yi, Zhanyuan Lu

**Affiliations:** 1 School of Life Science, Inner Mongolia University, Hohhot 010020, China; zhangfan98hl@126.com (F.Z.); liuying836354822@126.com (Y.L.); majie19952021@163.com (J.M.); 2 Inner Mongolia Academy of Agricultural & Animal Husbandry Sciences, Hohhot 010031, China; sushaofeng2020@163.com (S.S.); chenliyu1102@163.com (L.C.); yuchen_cheng@163.com (Y.C.); nmgbaater@163.com (S.B.); 3 Key Laboratory of Black Soil Protection and Utilization, Ministry of Agriculture and Rural Areas, Inner Mongolia Key Laboratory of Degradation Farmland Ecological Remediation and Pollution Control, Inner Mongolia Conservation Tillage Engineering Technology Research Center, Hohhot 010031, China; 4 Agricultural College, Inner Mongolia Agricultural University, Hohhot 010019, China

**Keywords:** expression profile, functional annotation, heat map, transcription factor, WGCNA

## Abstract

The MYB transcription factor family has numerous members, and is involved in biological activities, such as ABA signaling, which plays an important role in a plant’s resistance to abiotic stresses such as drought. However, the diversity of MYB members that respond to drought stress and their regulatory mechanisms in different flax varieties were unclear. In this study, we obtained 855.69 Gb of clean data from 120 flax root samples from 20 flax (*Linum usitatissimum* L.) varieties, assembled 92,861 transcripts, and identified 434 *MYB* family members in each variety. The expression profiles of the MYB transcription factor family from 20 flax varieties under drought stress were analyzed. The results indicated that there are four strategies by which the MYB family responds to drought stress in these 20 flax varieties, each of which has its own specific processes, such as development, reproduction, and localization processes. The four strategies also include common biological processes, such as stimulus responses, metabolic processes, and biological regulation. The WGCNA method was subsequently employed to identify key members of the MYB family involved in response strategies to drought stress. The results demonstrated that a *1R-MYB* subfamily gene co-expression network is significantly related to the gibberellin response and cytokinin-activated signaling pathway processes in the ‘Strategy 4’ for MYB family response to drought, identifying core genes such as *Lus.scaffold70.240*. Our results showed a diversity of MYB family responses to drought stress within flax varieties, and these results contribute to deciphering the mechanisms of the MYB family regulation of drought resistance. This will promote the more accurate breeding development of flax to adapt to agricultural production under drought conditions.

## 1. Introduction

Drought is one of the most significant abiotic stresses that affect plant growth and development, causing a series of reactions, such as the wilting of plant leaves, premature flowering, growth stagnation, or even death [1,2]. The MYB family of transcription factors (V-myb avian myeloblastosis viral oncogene homolog) is one of the largest families of transcription factors in plants [3,4,5,6]. According to the location and number of repetitive sequences in the MYB domain, MYB transcription factors can be divided into four subfamilies as follows: 1R-MYB (MYB-Related), 2R-MYB (R2R3-MYB), 3R-MYB, and 4R-MYB [7,8,9,10]. When subjected to drought stress, MYB transcription factors can play a role in conducting ABA signals, regulating the transcription of functional genes and other functions, and enhancing the drought resistance of plants [11,12]. The results of multiple studies support MYB as a potential transcription factor for plant breeding and improvement [13,14].

MYB transcription factors are involved in plant drought resistance in a variety of ways. Firstly, MYBs mainly depend on ABA signals to participate in the regulation of stomatal open–close movement [15]. For example, *MYB44* can prevent the inhibition of RCA 1/PYL9 on ABI1 phosphatase activity by binding to ABA receptors such as PYL8, PYL9, and so on, thus participating in ABA signaling [16]. *AtMYB77* can interact with *MYB44* to form a heterodimer to work together under drought stress, and the *myb44–myb77* double mutants have shown strong drought resistance [17]. The MYB transcription factor is also involved in the drought-resistant process associated with leaf permeability, by regulating the synthesis of flavonoids and the stratum corneum. The R2R3-MYB subfamily members of *Arabidopsis thaliana*, MIXTA-like genes *MYB16* and *MYB106*, can regulate the formation of the stratum corneum in trichomes [18]. The transcription factor *MYB12* in *Arabidopsis thaliana* is a key regulator of flavonoid biosynthesis, and the overexpression of *MYB12* and *MYB75* in transgenic plants significantly increases flavonoid accumulation, thus improving drought tolerance [19]. In addition, the drought resistance of the MYB family has also been widely described in other plants. For example, the expression of the *AtMYB68* gene in *Brassica napus* enhanced its drought resistance [20]. Under drought stress, the expression of the *TdMYB4A063* gene in durum wheat in the root system was significantly up-regulated after 2 h [21].

However, even for homologous genes, members of the MYB family have different expression patterns in the life activities of different species, and often even in different varieties of the same species. For example, the expression levels of transcription factors such as *MYB15* in response to cold stress were significantly lower in tea varieties with high cold tolerance than those of sensitive varieties [22]. This indicates that the differences in varieties also diversify the MYB gene expression profile.

Flax (*Linum usitatissimum* L.) can be categorized based on its development into fiber flax, oil flax, and oil–fiber flax. Oil flax is one of the most important oil crops in the world; it is rich in nutrients that are beneficial to human health, such as omega-3 fatty acids [23,24,25]. Flax has good drought tolerance, making it a relevant material for studying plant drought resistance [26]. In this study, by analyzing the expression profiles of MYB family members in 20 flax varieties under drought stress, we identified combination patterns between the biological processes that MYB families participate in under drought stress. And then we summarized the response strategies of MYB members of different flax varieties to drought stress, and dug out the preference of MYB family members in these strategies. These results have significant reference value for shedding light on the cooperative drought resistance mechanism among members of the MYB family, and improving the efficiency of molecular breeding for drought resistance in crops such as flax.

## 2. Results

### 2.1. Flax MYB Family Member Distribution

By screening the genes containing the MYB domain in the amino acid sequence of the flax genome, 434 MYB genes were obtained, among which 235 genes were members of the 1R-MYB subfamily, 194 genes were members of the 2R-MYB subfamily, 3 genes were members of the 3R-MYB subfamily, and 1 gene belonged to the 4R-MYB subfamily. In addition, the *Lus.scaffold127.115* gene contained five MYB domains. The gene IDs and domains of the MYB family members are given in Supplementary Table S1.

### 2.2. Quality Control of the Transcriptome Sequencing of 20 Flax Varieties

This sequencing involved three biological replicates of 20 flax varieties in two treatment groups (drought group and control group), with a total of 120 samples, from which 855.69 Gb of clean data were obtained, and 92,861 transcripts were assembled. The average error rates of the sequenced bases corresponding to data from each sample ranged from 0.0251% to 0.0268%, that of Q20 ranged from 97.33% to 97.98%, that of Q30 ranged from 92.61% to 94.11%, and that of GC content ranged from 45.99% to 49.65% (Supplementary Table S2).

### 2.3. Expression Levels and Difference Analysis of the MYB Family

**Figure 1:**
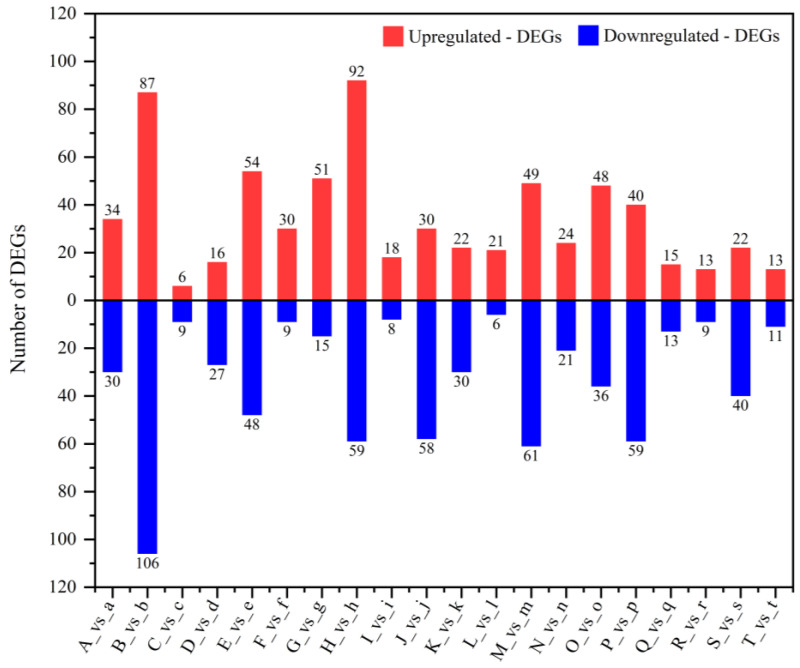
Distribution diagram of DEGs of the MYB family in 20 flax varieties.

DEGs in the MYB family of 20 flax varieties were screened and counted (Table 1, Figure 1). The results showed that all varieties had members of the MYB family that were differentially expressed under drought stress. Variety B (Zhangya No.1) had the most DEGs in the MYB family (193). In addition, the number of DEGs in the MYB families of the M (Ningya No.19, 110), H (Dingxi No.17, 151), E (Yiya No.4, 102), and P (Baya No.15, 99) varieties was also high (≥99), suggesting that the members of the MYB family played a larger role in the process of drought resistance within these varieties. The number of DEGs in the MYB family of variety C (Ningya No.15) was the least (15). In addition, the numbers of DEGs in the MYB family of R (Longya No.10, 22), T (Neiya No.9, 24), I (BGOLDXREDWING44X3, 26), L (Ningya No.17, 27), and Q (Gaolanbai, 28) were also small (<30).

Among the 20 flax varieties selected in this study, only A (Longza No.1) and T (Neiya No.9) were water-sensitive varieties. A’s (Longza No.1) MYB family had 64 DEGs, which was considered a medium number among the 20 flax varieties. The MYB family of T (Neiya No.9) had only 24 DEGs, which is considered small; thus, it was speculated that the expression level of the MYB family might affect the drought resistance of T (Neiya No.9).

### 2.4. The Diversity of Expression Patterns 

**Figure 2:**
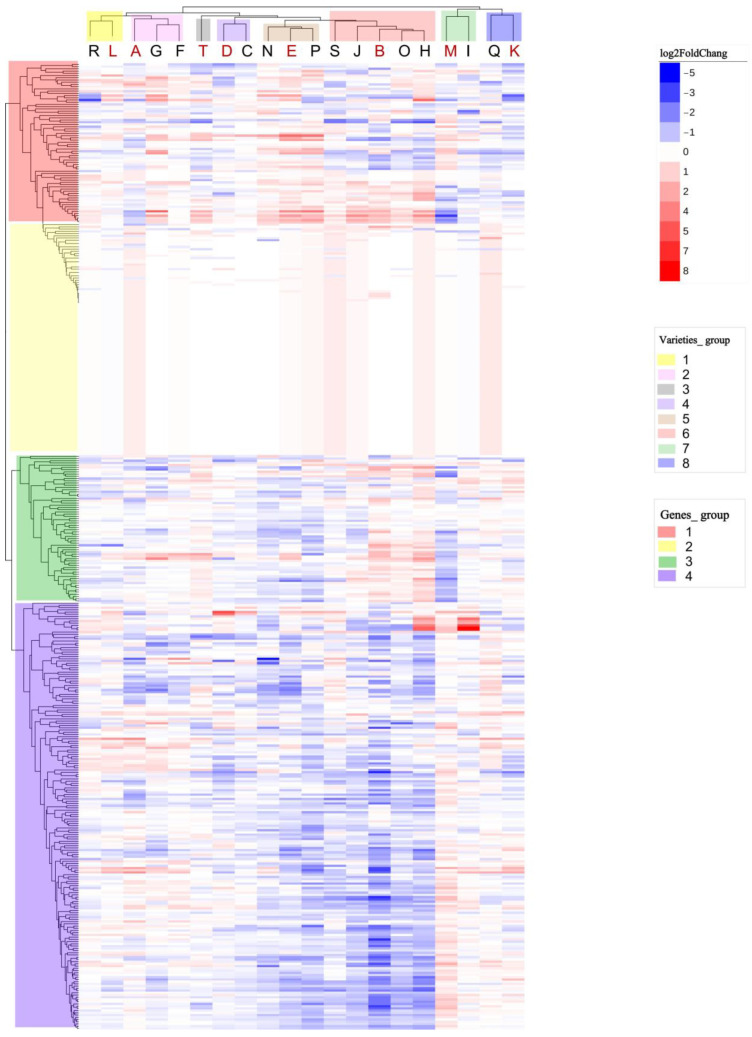
Cluster analysis of the expression levels in the drought group of 20 flax varieties. Each row in the figure represents a variety, and each column represents an MYB gene. The color in the heat map represents the value of the log2FoldChange. On the left is the tree diagram of gene clustering, and on the top is the tree diagram and variety code of sample clustering. Varieties marked in red were involved in the subsequent functional analysis of this study, and the color of the tree represents their clustering grouping.

The difference in the expression patterns of MYB family members in different varieties may have reflected the diversity of their regulatory pathways. According to the expression difference (log2FoldChange) of multiple MYB family members in the drought group and the control group in each flax variety, the MYB family distance of different flax varieties was calculated, hierarchical clustering was performed on the flax varieties, and a heat map was drawn (Figure 2). According to the clustering results, the MYB family expression patterns of the 20 flax varieties were divided into eight groups, and all MYB genes in the flax genome were divided into four groups.

In the expression pattern clustering results, R (Longya No.10) and L (Ningya No.17) were clustered into Group 1. A (Longza No.1), G (Zhangya No.2), and F (Dingya No.15) were clustered into Group 2, and T (Neiya No.9) was not clustered with other varieties, thus becoming Group 3 alone. D (Longya No.8) and C (Ningya No.15) were clustered into Group 4, and N (Baxuan No.3), E (Yiya No.4), and P (Baya No.15) were clustered into Group 5. S (Yiya No.5), J(R43), B (Zhangya No.1), O (Sha Che Zao Shu Zhong Hong), and H (Dingxi No.17) were clustered into the largest group in this variety clustering, Group 6. A large number of flax varieties in this group were down-regulated in Group 4 of the MYB gene clustering. Varieties M (Ningya No.19) and Q (Gaolanbai) were in the seventh group, while I (BGOLDXREDWING44X3) and K (Lixian) were in the eighth group (Figure 2).

### 2.5. Analysis of Potential Biological Significance of Expression Patterns

According to the expression value clustering of MYB family members, the 20 flax varieties were divided into eight groups (Figure 2). Next, we selected one flax variety from each group as the representative, and conducted the functional annotation of its DEGs in the MYB family to explore the underlying biological significance of different expression patterns. In addition to the water-sensitive variety A (Longza No.1, 64 DEGs) in the second group and the water-sensitive variety T (Neiya No.9, 24 DEGs) in the third group, the varieties with the most DEGs were selected in other groups in this study, including L (Ningya No.17, 27 DEGs), D (Longya No.8, 43 DEGs), E (Yiya No.4, 102 DEGs), B (Zhangya No.1, 193 DEGs), M (Ningya No.19, 110 DEGs), and K (Lixian, 52 DEGs). The KEGG annotations (Table 2) and GO analysis (Table 3 and Supplementary Table S3) were performed on DEGs of the MYB family in these eight varieties, and the response strategies of the MYB family to drought stress in different flax varieties were summarized by analyzing the combination patterns of biological processes annotated by MYB family DEGs in different flax varieties.

The KEGG annotation of the MYB family DEGs was performed on the eight flax varieties, and the results showed that *Lus.scaffold440.7*, a member of the flax 2R-MYB subfamily, was annotated with “Pentose and glucuronide conversions” under the “Metabolism category” in five flax varieties (A, D, E, L, T). *Lus.scaffold111.105*, a member of the 1R-MYB subfamily, was annotated with the “Circadian rhythm-plant” pathway in four varieties (B, K, L, M). “Plant Hormone Signal Transfer” was a unique KEGG pathway of variety B (Zhangya No.1), with which three 1R-MYB subfamily members (*Lus.scaffold70.63*, *Lus.scaffold45.334* and *Lus.scaffold34.70*) were annotated (Table 3, Supplementary Table S1).

The GO molecular functions annotated for the MYB families of the eight varieties included transcription regulator activity and binding. The greater the number of DEGs present in the varieties, the more obvious the gene amplification of the binding function. In addition, the catalytic activity in molecular functions was annotated by DEGs within the MYB family of seven flax varieties (A, T, L, D, E, B, and M), indicating the presence of MYB transcription factors with catalytic activity in multiple flax varieties (Table 2 and Supplementary Table S3).

The GO annotation of biological processes showed that the biological processes involved in all eight varieties included the response to stimulation, metabolic processes, and biological regulation. These biological processes were a common element of the strategies of the MYB family in responding to drought stress across all eight flax varieties. The MYB family response to drought stress in the eight groups was divided into four strategies, based on specific biological processes where the annotated DEGs were not present in all varieties. The groups represented by the D and L varieties were categorized as ‘Strategy 1’, and did not contain specific biological processes. The group represented by the T variety was ‘Strategy 2’, and the unique biological process was the developmental process. The group represented by A, E, and K was ‘Strategy 3’, and the specific processes were the developmental process, cellular component organization or biogenesis, the reproductive process, and growth. The group represented by varieties B and M was categorized as ‘Strategy 4’, and the unique biological processes were the developmental process, cellular component organization or biogenesis, the reproductive process, growth, and the localization process. In each strategy, common biological processes and unique biological processes were combined into a combination module able to respond to drought stress.

### 2.6. MYB Family Co-Expression Network and Core Drought Resistance Members

Among the four strategies of the MYB family of the 20 flax varieties in response to drought stress, ‘Strategy 4’ had the most biological processes annotated by DEGs, suggesting that this strategy is more likely to require coordination and interaction among MYB family members. Therefore, according to the expression of MYB family members and the results of the functional annotations, we selected the five following flax varieties belonging to Strategy 4 of the MYB family response to drought stress: S (Yiya No.5), J (R43), B (Zhangya No.1), O (Sha Che Zao Shu Zhong Hong), and H (Dingxi No.17). We took them as an example to mine the co-expression module of MYB family members in Strategy 4 in response to drought stress, and mined the preferred MYB gene of this strategy. There were three biological replicates for each variety in the drought and control groups, resulting in a total of 30 samples. The gene co-expression modules and preferred genes of the flax MYB family related to drought resistance were screened and identified based on the WGCNA (weighted gene co-expression network analysis) algorithm.

**Figure 3:**
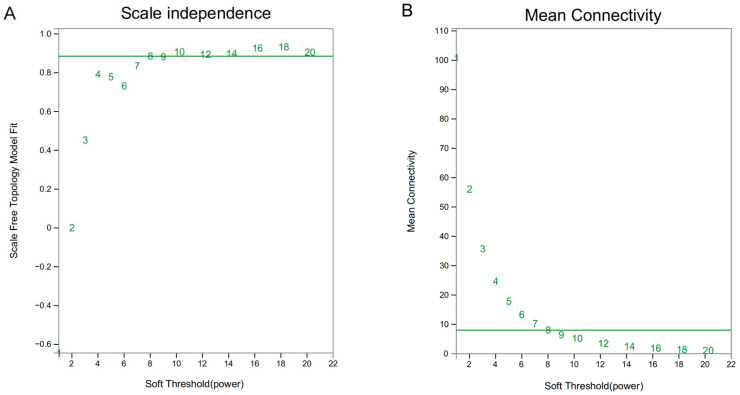
The scaling free curve of the gene co-expression module. (**A**): no scale tolerance curve, (**B**): scale-free mean connectivity curve.

After the background correction and standardization of the expression data of the MYB family members, abnormal and small-mutation genes were filtered. Subsequently, 255 genes exhibiting a related action intensity, most in line with the scale-free distribution condition, were selected for analysis from the 434 MYB family members. The soft threshold of β = 8 was selected for module identification and gene co-expression network construction, because the scale-free network reached a value above 0.9 for the first time in these conditions, indicating good connectivity of the co-expression network (Figure 3).

**Figure 4:**
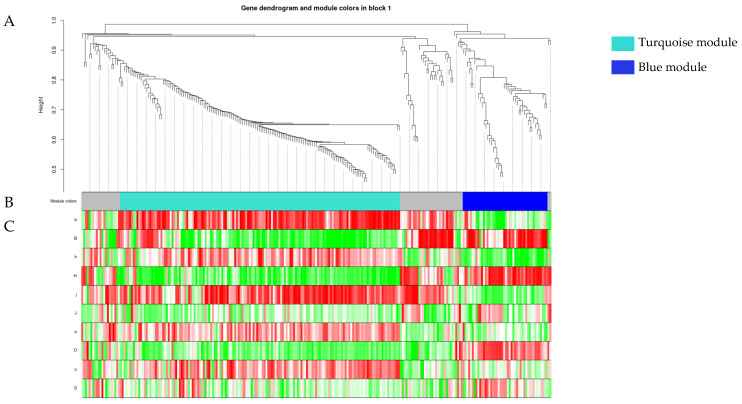
Flax MYB family WGCNA module classification tree. The top half of the figure (**A**) is a gene-level cluster tree, with each branch representing an MYB gene. The middle part of the figure (**B**) is the co-expression module, to which this MYB gene belongs. Each color represents a module, and gray modules represent genes that were not divided into specific modules. The lower part of the figure (**C**) is a heat map of the correlation between this MYB gene and the sample. Each row represents a set of treatments for a flax variety. The darker the color, the greater the correlation between this MYB gene and the sample, with red indicating a positive correlation and green indicating a negative correlation.

The blockwiseModules function was used for module identification and module analysis (Figure 4). In this study, a total of two co-expression modules, ‘Turquoise’ and ‘Blue’, were identified in Strategy 4. There were 152 MYB genes in the ‘Turquoise’ module, and 46 MYB genes in the ‘Blue’ module. The results showed that most genes in the ‘Turquoise’ module had a positive correlation with the control samples (b, h, j, o, and s) and a negative correlation with the drought samples (B, H, J, O, and S), while most genes in the ‘Blue’ module had a positive correlation with the drought-treated samples (B, H, J, O, and S) and a negative correlation with the control samples (b, h, j, o, and s). Therefore, it was considered that most genes in the ‘Turquoise’ module were down-regulated in expression under drought stress, while most genes in the ‘Blue’ module were up-regulated in expression under drought stress.

**Figure 5:**
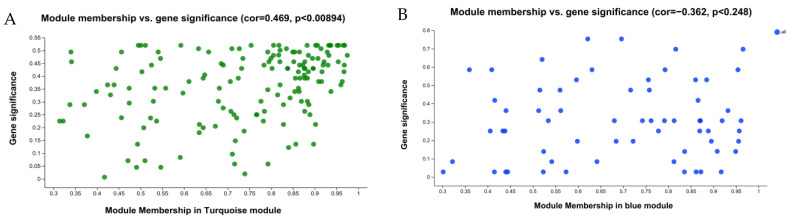
Group 6 flax variety MM-GS scatter plot. (**A**): Turquoise module MM-GS scatter diagram; (**B**): Blue module MM-GS scatter diagram. Module membership (MM): also known as kME (eigengene-based connectivity) correlation between the expression pattern of a gene and module characteristic genes; gene significance (GS): a measure of the importance of a gene.

The MM-GS scatter plots of the ‘Turquoise’ and ‘Blue’ modules were constructed (Figure 5). The results showed that, in the ‘Turquoise’ module, there was a significant correlation between GS and MM (cor = 0.469, *p* < 0.00894), while in the ‘Blue’ module, the correlation between GS and MM was low (cor = −0.25, *p* < 0.183). Therefore, we judged that there were more genes in the ‘Turquoise’ module that were highly correlated with the samples. And there were also more MYB family members within its modules that were highly correlated with drought resistance. Therefore, we further annotated and enriched the MYB family members in the ‘Turquoise’ module.

**Figure 6:**
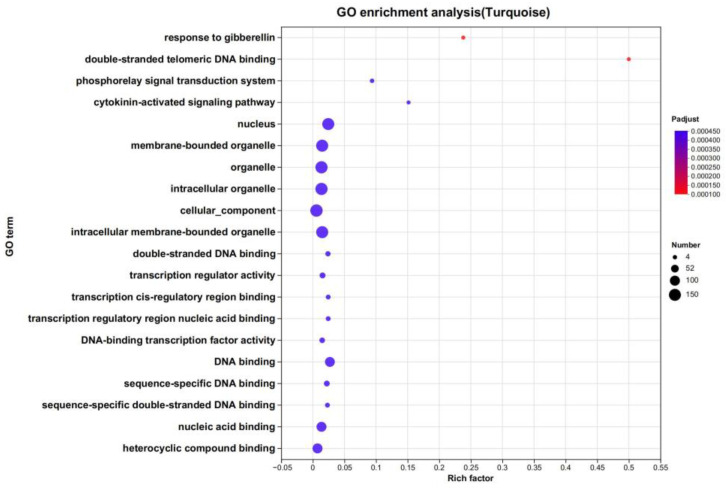
GO enrichment bubble diagram of the ‘Turquoise’ module. The vertical axis represents the GO Term, and the horizontal axis represents the Rich factor, which is the ratio of enriched genes to annotated genes in that term. The size and color of each dot indicate the number of genes and *p*-adjust ranges, respectively. Only results with a *p*-adjust < 0.5 are displayed for the top 20 enrichments.

All MYB family members in the ‘Turquoise’ module were annotated and enriched for GO analysis, and a GO enrichment bubble map was constructed (Figure 6). The results showed that some genes were significantly enriched in the gibberellin response and the signaling pathway processes of cytokinin activation. Therefore, the ‘Turquoise’ module could conduct signal transduction by participating in the process of the response to plant hormones, thereby helping flax varieties resist drought stress.

**Figure 7:**
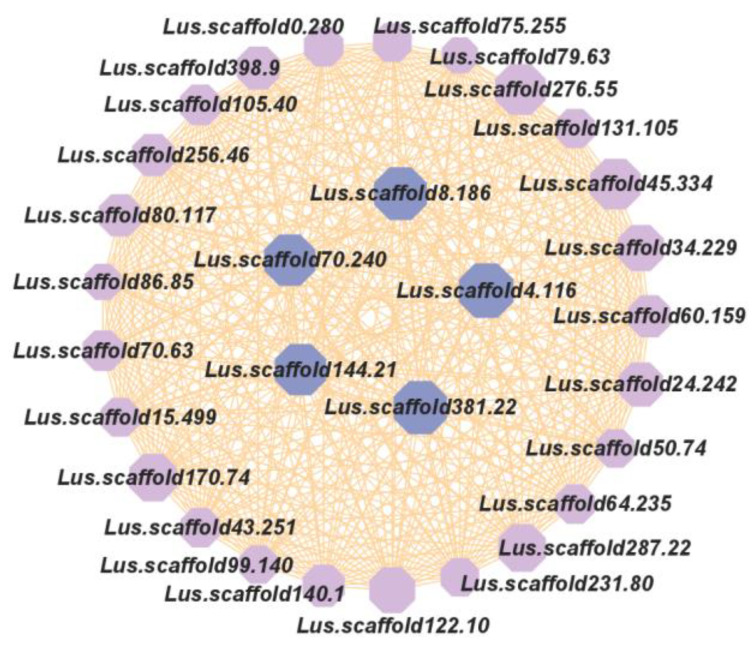
Turquoise module gene co-expression network. Each node in the figure represents an MYB gene and is labeled with the gene ID. The size of a node’s circle represents its degree of connectivity. The larger the circle, the higher the degree of connectivity and the more radiation edges. The inner circle represents the five nodes with the highest connectivity.

The gene nodes in the top 30 for node connectivity (degree) in the ‘Turquoise’ module were screened to construct a gene co-expression network (Figure 7). Located in the inner circle were the five genes with the highest node connectivity in the module. The five MYB family members in the inner ring (*Lus.scaffold4.116*, *Lus.scaffold381.22*, *Lus.scaffold8.186*, *Lus.scaffold144.21*, *Lus.scaffold70.240*) belonged to the 1R-MYB subfamily, where the gene *Lus.scaffold70.240* was a homologous gene of *AtMYB88*, a member of the *Arabidopsis thaliana* MYB family (Supplementary Table S5). Therefore, these five genes play an important role in the ‘Turquoise’ module, and belong to the core genes of the module. Based on this, these five genes are the preferred genes of the flax MYB family in response to drought stress ‘Strategy 4’.

**Figure 8:**
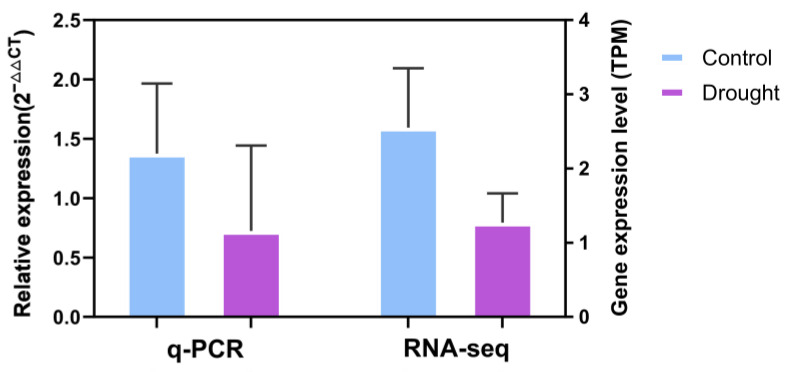
Expression levels of *Lus.scaffold70.240* of the S (Yiya No.5) variety in the control group and the drought treatment group in q-PCR experiments and transcriptome sequencing. The left *Y*-axis represents the expression of this gene in the control group and the drought group in the q-PCR experiment, and the expression value is calculated by 2^−∆∆CT^ method; the right *Y*-axis represents the expression of this gene in the transcriptome sequencing in the control group and the drought group, and the expression level is expressed by the TPM value.

In order to clarify the expression condition of the ‘Strategy 4’ preference gene, which serves as the core gene of the ‘Turquoise‘ module, and to verify the accuracy of the transcriptome data, we selected variety S (Yiya No.5) (Figure 2, Table 4) to represent the flax variety following ‘Strategy 4’. Subsequently, q-PCR experiments were conducted using the *Lus.scaffold70.240* gene, which is a homologous gene of *Arabidopsis thaliana AtMYB88*, as an example (Figure 8, Supplementary Table S5). The results showed that the expression trend of *Lus.scaffold70.240* gene was consistent in q-PCR and transcriptome sequencing (Figure 8). The expression of this gene was down-regulated under drought stress, which was also consistent with our conclusion that most genes in this module were down-regulated under drought stress by drawing the heat map (Figure 4) of the ‘Turquoise’ module.

## 3. Discussion

The inability of plants to move leads to the need to mobilize gene expression in order to resist the growth retardation and oxidative damage caused by a drought environment, in which the transcription-factor-mediated gene expression regulatory network plays an important role [27,28]. The MYB transcription factor family is one of the largest transcription factor families in plants, and plays an important role in regulating plant organ development, secondary metabolism, the hormone signaling network, the cell cycle, and other biological processes during drought stress [29,30]. In this study, we obtained the transcriptome data of the MYB family in 20 flax varieties through field water treatment experiments. By analyzing the expression profiles of the MYB family under drought stress, we summarized the four strategies of the MYB family in these 20 flax varieties in response to drought stress and the preferred MYB family members in Strategy 4. This study enabled us to understand the combination models of the flax MYB transcription factor family that regulate biological processes under drought stress, and will help us to decode the synergistic drought resistance mechanism of MYB family members in the future. This will allow us to improve the efficiency of molecular breeding for the drought resistance of flax and other crops.

### 3.1. The Distribution of DEGs in the MYB Family of 20 Flax Varieties Was Significantly Different

Because different varieties have different paternal and maternal parents, and have been subjected to selection pressures from different places of origin, there is a high degree of genetic diversity between the genomes of different varieties of the same species [31]. This genomic diversity indicates that the expression patterns of different varieties of the same species will also be diverse under different growth stages or abiotic stress conditions [32]. A large number of transcriptome analysis results support this claim [33,34,35,36]. For example, the expression profiles of two potato varieties with contrasting drought tolerance showed significant differences during both the early and late stages of drought stress, as well as after rehydration [34]. Among various sorghum varieties, drought stress induced distinct changes in the expression patterns of many homologous genes related to cutin monomer and cutin wax biosynthesis [35]. 

In our previous studies, we confirmed that there is a wide genetic diversity in the genomes of different varieties of flax, which is influenced by many factors, such as parental and geographical origin [37]. In this study, we confirmed that the distribution of DEGs in the MYB family of different flax varieties is also significantly different under drought stress (Table 1, Figure 1). For example, there was a large gap between the 20 varieties in the number of DEGs (15~193). Five varieties (Zhangya No.1, Ningya No.19, Dingxi No.17, Yiya No.4, and Baya No.15) had more DEGs in the MYB family (≥99), while another five varieties (Ningya No.15, Longya No.10, Neiya No.9, Ningya No.17, Gaolanbai, and BGOLDXREDWING44X3) had fewer DEGs in the MYB family (<30) (Table 1, Figure 1).

### 3.2. The Response Strategies of the MYB Family to Drought Stress Were Diverse among Different Flax Varieties

The first step in exploring MYB’s response strategies to drought stress is to clarify the expression of MYB genes under drought stress. Similar expression patterns in the MYB family will correspond to similar strategies in response to drought stress [38]. In order to further clarify the similarity of the expression patterns of the MYB family in 20 flax varieties, we clustered the 20 flax varieties based on changes in the expression levels (log2FoldChange) of MYB family members under drought stress (Figure 2). The results showed that the 20 flax varieties could be divided into eight groups, and the expression patterns of the MYB family members in each group were similar (Figure 2).

Since the expression patterns of the flax MYB family in each group were similar, we screened the expression patterns of the flax MYB family in each group under drought stress, functionally annotating the DEGs. The KEGG annotation results showed that *Lus.scaffold440.7*, a member of the 2R-MYB subfamily of flax, was annotated with pentose and glucuronic acid conversion pathways under the metabolism category in five flax varieties (A, D, E, L, and T) (Table 2). The impact of drought stress on the expression of genes related to sugar metabolism has also been reflected in a large number of previous studies. For example, following drought stress, the genes related to starch synthesis in tea trees were down-regulated, while those related to starch decomposition were up-regulated, with 74 DEG annotations in the pentose and glucuronic acid conversion pathways [39]. The *MYB28*, *MYB29*, and *MYB76* transcription factors have been identified as important regulatory factors of Aliphatic Glucosinolate (AG) biosynthesis [40,41,42]. The transcription factors *MYB34*, *MYB51,* and *MYB122* are important regulators of Indolic Glucosinolate (IG) biosynthesis [43,44].

According to the biological processes in the GO functional annotation of the DEGs in the MYB family, eight varieties could be classified into four strategies in response to drought (Table 3). The common biological processes in these four strategies included the response to stimulation, metabolic processes, and biological regulation. These biological processes have been proven by a large number of studies to be the basic regulatory processes initiated by transcription factors under drought stress [45,46,47]. The unique biological process of ‘Strategy 2’ was the developmental process. The functional prediction results of the MYB family of *Cajanus cajan* also showed that *CcR2R3-MYBs* was involved in the developmental process and responded to abiotic stress, and the overexpression of *CcMYB107* significantly improved the drought resistance of plants [48]. The specific processes of ‘Strategy 3’ were the reproductive process and growth. Nine *R2R3-MYB* members that were constitutively expressed in male reproductive tissues were also identified in Chinese cabbage, based on organ-specific expression data [49]. The biological processes specific to ‘Strategy 4’ were localization and others. TvCyP1 cyclophilin has been proven to regulate the nuclear translocation of Myb1 and Myb3. It can directly bind to Myb3, and it can mediate a series of the conformational switches of Myb3 from the membrane chamber to the nucleus [50]. The pathways by which the MYB family regulates drought resistance have been extensively studied, and our work identified the pattern of the combinations of these biological processes involved in the regulation of the MYB family in the 20 flax varieties [44,51]. In the future, we will explore which model of the MYB family is the most efficient in regulating drought resistance, which will have important reference value for the breeding work of flax and other crops.

In this study, *Lus.scaffold440.7* (2R-MYB subfamily), a homolog of the *AtMYB93* gene, was simultaneously annotated in two GO terms (metabolic process and catalytic activity function) in five flax varieties (A, D, E, L, and T). Therefore, it was speculated that this gene product had catalytic activity, and could play a catalytic role in the metabolic process of multiple flax varieties (Supplementary Table S1). Several studies have shown that *AtMYB93* is a negative regulator of lateral root development in *Arabidopsis thaliana*; it is strongly expressed in early lateral root endodermal cells, and it is induced by auxin in the meristem of the primary root [52,53]. In the future, we plan to conduct more detailed bioinformatics and molecular biological analyses of this gene in order to determine how effective it is in improving the drought resistance of different flax varieties.

### 3.3. WGCNA Mines the Preferred MYB Family Members of Strategy 4 Such as Lus.scaffold70.240

In this study, we found a *1R-MYB* subfamily gene co-expression module (‘Turquoise’ module) that was highly related to drought resistance in Strategy 4, and most of the genes in the module were down-regulated under drought stress (Figure 4). The GO annotation and enrichment results indicated that some genes in this module were significantly enriched in the gibberellin response process and the cytokinin activation signaling pathway (Figure 6). Research has shown that the *NtMYB330* transcription factor in tobacco can regulate the expression of genes related to abscisic acid (ABA) and gibberellic acid (GA) signaling, affecting both ABA-GA crosstalk and seed germination [54]. Studies have shown that the transcription of cytokinin target genes is regulated by type-b response regulatory proteins (RRBs), a transcription factor of the MYB family [55,56]. In addition, a transgenic experiment on the *VyMYB24* gene of a highly drought-resistant grape variety showed that the gene may inhibit plant development by regulating gibberellin (GA) metabolism [57]. 

WGCNA screened the five following genes, which had the highest connectivity in this module: *Lus.scaffold4.116*, *Lus.scaffold381.22*, *Lus.scaffold8.186*, *Lus.scaffold144.21*, and *Lus.scaffold70.240* (Figure 7). In the qRT-PCR experiment, the expression of the *Lus.scaffold70.240* gene was down-regulated under drought stress, which was consistent with the trend of transcriptome sequencing (Figure 8). The *Lus.scaffold70.240* gene is a homologue of the *AtMYB88* gene of *Arabidopsis thaliana*, and the *AtMYB88* gene can regulate the late division of stomatal cell lineage [58]. qRT-PCR showed that *PsFLP*, the homologous gene of *AtMYB88* in *Pisum sativum*, was involved in the response of *Pisum sativum* to drought stress [59]. *Lus.scaffold4.116 and Lus.scaffold144.21* are the homologous genes of the *Arabidopsis thaliana* MYB family TBP-like type genes—*AT1G07540* and *AT3G12560* (Supplementary Table S5)—which play an important role in regulating the structure and function of eukaryotic telomeres. Severe drought stress can cause DNA damage in plants [60]. RT-PCR experiments showed that some genes encoding TBP (Telomeric DNA-binding proteins), such as *AtTRP2*, were down-regulated when the *Arabidopsis thaliana* DNA was damaged [61]. This result is consistent with the conclusion of this study that most genes in the ‘Turquoise’ module are down-regulated under drought stress, which can confirm the conclusion of this study (Figure 4). Due to the highest connectivity, we speculated that these five genes are the preferred genes in the MYB family of flax for coping with drought stress under ‘Strategy 4’. These five genes will be the focus of our future research. We will use a large number of molecular biology experiments, such as q-PCR, sub-cellular localization, immunoprecipitation, gene knockout, etc., to explore the specific biological processes in which these genes participate in regulation under drought stress, as well as their impact on crop drought resistance. This will have important reference value for the molecular breeding of crops such as flax.

## 4. Materials and Methods

### 4.1. Identification of MYB Family Members

HMMER-3.0 software [62] was used to identify the MYB domain (PF00249) in the flax amino acid sequence. The criteria for determining the characteristics of the conservative domain were as follows: (1) The coefficient of similarity with the confirmed conservative sequence of the MYB domain, or conditional E-value value, was less than 1 × 10^−5^; and (2) the conservative sequence comprised at least one conservative tryptophan (W) [63]. ID simplification was performed on the obtained genes, and the genes obtained after the deletion of repeat sequences according to the GFF files were counted as members of the MYB family. According to the number of conservative domains of MYB, it was divided into four subfamilies as follows: 1R-MYB (MYB-Related), 2R-MYB (R2R3-MYB), 3R-MYB, and 4R-MYB [63,64]. The flax amino acid sequence, GFF, and other data were obtained from the Figshare database (https://figshare.com/articles/dataset/Annotation_files_for_Longya-10_genome/13614311, accessed from 15 October 2022).

### 4.2. Plant Materials

Based on previous research, such as the identification and cluster analysis of drought resistance in early field experiments, combined with the recommendations of drought-resistant varieties from the national oil crop industry technical system, 18 drought-resistant flax varieties and two water-sensitive varieties as control varieties were screened in this study to conduct the drought stress test (Table 4, Supplementary Table S4) [37].

### 4.3. MYB Gene Expression Induction Method

In this experiment, a split-plot design was adopted, and the main treatments were the two following kinds of water treatments: drought treatment and control treatment. The secondary treatment included 20 flax varieties, of which 18 were drought resistant and two were water sensitive. Each variety of each treatment had three repeats.

The 20 flax varieties grew under natural conditions in the field until the water-sensitive varieties grew to 20 cm, at which time, a dry shed was set up for the water treatment, and all the flax varieties had entered the full bloom stage. The water treatment was set as two gradients, which were divided into (1) control treatment (the soil water content was maintained at 70~75% of the maximum field water holding capacity) and (2) drought treatment (the soil water content was maintained at 25~30% of the maximum water holding capacity).

**Figure 9:**
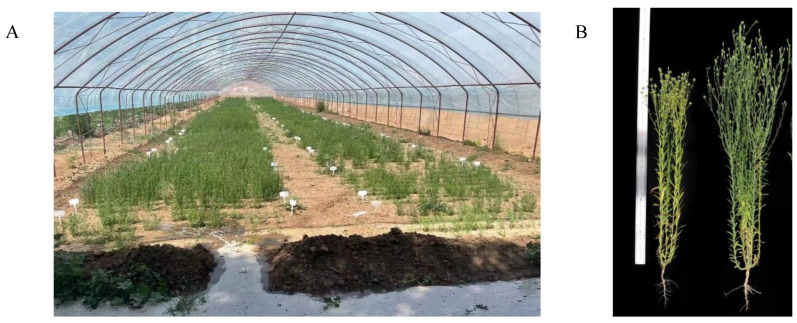
The drought shed and plants used in the experiment. (**A**): flax moisture treatment test in the dry shed; (**B**): flax plants during transcriptome sampling. The plant on the left is the Zhangya No.1 variety under drought treatment, and the plant on the right is a sample of Zhangya No.1 under control treatment.

When the water treatment reached a significant level, where 80% of the water-sensitive varieties wilted or even lodged under drought treatment, and about 50% of the varieties in the drought-resistant group exhibited leaf wilting (at this time, the water treatment had lasted for 30 days, Figure 9), the roots of the flax were sampled, and three replicates were randomly selected from each group, totaling 120 samples (20 varieties × 2 treatments × 3 replicates). The samples were immediately frozen in a −80 °C refrigerator for cryopreservation until transcriptome sequencing at Majorbio company (Beijing, China). At the same time, we also took three samples of variety S (Yiya No.5) in the control group and treatment group (1 varieties × 2 treatments × 3 replicates) for the qRT-PCR experiment to verify the transcriptome data.

### 4.4. Determination and Analysis of the MYB Gene Expression Level

#### 4.4.1. RNA Extraction

The total RNA in the samples was obtained via the Invitrogen Method, and genomic DNA was removed using DNase I (TaKara, Kyoto, Japan) [65]. The concentration and purity of the extracted RNA were detected using NanoDrop 2000 (NanoDrop Technologies, Wilmington, NC, USA). RNA integrity was detected using agarose gel electrophoresis, and the RIN value was determined by Agilent 2100 (Agilent Technologies, Santa Clara, CA, USA) to ensure the transcriptome sequencing was performed using qualified samples (OD 260/280 = 1.8–2.2, OD260/230 ≥ 2.0, RIN ≥ 6.5, 28S: 18S ≥ 1.0, >1 μg).

#### 4.4.2. cDNA Library Construction and Computer Sequencing

The RNA library was constructed using the TruseQ^TM^ RNA Sample Preparation Kit (IILUMINA, San Diego, CA, USA). MRNAs were first isolated and enriched from the total RNA by A-T base pairing with Oligo(dT)-bearing magnetic beads with the 3′ terminal ployA of mRNA. The mRNA was randomly fragmented by adding fragmentation buffer, and small fragments of about 300 bp were isolated via magnetic bead screening. Next, using a Superscript Double-Stranded cDNA Synthesis Kit (Invitrogen, Carlsbad, CA, USA), six-base random hexamers (IILUMINA, San Diego, CA, USA) were added, and mRNA was used as the template to invert and synthesize one-strand cDNA, followed by two-strand synthesis in order to form a stable double-strand structure. The double-stranded cDNA had a sticky end. End Repair Mix was added to make it a flat end, followed by an “A” base at the 3′ end for a connection to the Y-junction. After the enrichment of the cDNA by PCR, DNA Clean Beads screened the 200–300 bp band. After quantification via TBS380 (PicoGreen) (Molecular Probes, Eugene, OR, USA), the library was high-throughput-sequenced using the Illumina NovaSeq 6000 sequencing platform with a sequencing read length of PE150.

#### 4.4.3. Transcriptome Data Quality Control

Fastp-0.19.5 software [66] was used to remove the adapter sequence from the reads, and remove the reads without the inserted fragments due to linker self-ligation, etc. We pruned low-quality sequences with a 5′ end mass value of less than 20 and a 3′ end mass value of less than 3, and removed reads containing ambiguous bases. Sequences with a length of less than 30 bp after the removal of the adapter and mass pruning were discarded.

#### 4.4.4. Analysis of MYB Family Gene Expression

TPM transformation was performed on the Read Counts of each sample gene to obtain standardized gene expression levels. Subsequently, the expression levels of MYB family genes in all varieties and treatments were extracted to form an MYB family genes expression matrix, and expression difference analysis was performed using the DESeq2- 1.24.0 package in R language to calculate the differential expression multiple log2FoldChange of each variety in the drought group and the control group. The DEGs (differentially expressed genes) of the MYB family in each variety under drought stress were identified using *p*-value < 0.05, |log2FoldChange| > 1.

#### 4.4.5. Expression Pattern Cluster Analysis

The expression pattern clustering analysis was conducted based on the log2FoldChange matrix of the MYB genes of all 20 varieties using RSEM-1.3.3 software [67]. The clustering method of the genes and varieties was hierarchical clustering, and the clustering algorithm was Spearman. The expression patterns of the 20 flax varieties were grouped according to the variety clustering results. The MYB family DEGs of one variety for each group were selected for functional annotation, including GO annotation and KEGG annotation, to identify the functional diversity of the MYB family DEGs in different varieties.

#### 4.4.6. Analysis of the Gene Co-Expression Network

A WGCNA (weighted gene co-expression network analysis) algorithm was used to construct a gene co-expression network, and the code was written with reference to the WGCNA-1.63 package in R language for analysis [68]. The specific analysis process was as follows:

- Data preprocessing. Background correction and standardization were performed on the MYB gene expression data, and the genes with abnormal and small variations were filtered. Then, sample clustering and scale-free curve analysis were performed to determine the soft threshold β of the recognition module.
- Module identification. Module identification was performed using the blockwiseModules function, with the parameters set to minModuleSize = 30, minKMEtoStay = 0.3, and mergeCutHeight = 0.25.
- Module analysis. A target module that was highly correlated with the drought resistance of flax was found, and the genes in this module were analyzed for functional enrichment by using the Goatools-0.6.5 software. The analysis method was Fisher’s exact test, *p*-adjust < 0.05.
- Mining core genes of the target module. The degree of the connectivity of each node in the target module was calculated using Cytoscape-3.9.0 software, and the top 30 nodes were screened to construct a gene co-expression network [69].

#### 4.4.7. qRT-PCR Experiment for Validation

Primer-5 software was used to design the primer sequence, and the output production was 150–300 bp. The *Actin* gene of flax was used as the internal reference, and the primers and target gene sequences were shown in Supplementary Table S6. The reaction system was SYBR Premix Ex Taq (Tli RNaseH Plus) (2×) 10 μL, PCR Forward Primer 0.5 μL, PCR Reverse Primer 0.5 μL, ROX Reference Dye or Dye (50×) 0.4 μL, cDNA solution 1 μL, ddH_2_O 7.6 μL. The PCR procedure was 95 °C 30 s; 95 °C 5 s; 60 °C 31 s for a total of 40 cycles. Three biological replicates and three technical replicates were set up, and the relative gene expression was calculated using 2^−∆∆CT^.

## 5. Conclusions

This study investigated the expression profile diversity of the MYB transcription factor family in 20 flax varieties subjected to drought stress. The results indicated that there are four strategies by which the MYB family responds to drought stress in 20 flax varieties, each of which has its own specific processes, such as development, reproduction, and localization processes. The four strategies also involve common biological processes, such as stimulus responses, metabolic processes, and biological regulation. A *1R-MYB* subfamily gene co-expression network that was significantly related to the gibberellin response and the cytokinin-activated signaling pathway processes was identified in the ‘Strategy 4’ for MYB response to drought, and core genes such as *Lus.scaffold70.240* were obtained by WGCNA. In conclusion, the response strategies of the MYB families of different flax varieties to drought stress are diverse.

## Figures and Tables

**Table 1:** Quantity distribution of the DEGs of 20 flax varieties.

Variety Code	DEGs	Sig-Up	Sig-Down	Variety Code	DEGs	Sig-Up	Sig-Down
A_vs_a	64	34	30	K_vs_k	52	22	30
B_vs_b	193	87	106	L_vs_l	27	21	6
C_vs_c	15	6	9	M_vs_m	110	49	61
D_vs_d	43	16	27	N_vs_n	45	24	21
E_vs_e	102	54	48	O_vs_o	84	48	36
F_vs_f	39	30	9	P_vs_p	99	40	59
G_vs_g	66	51	15	Q_vs_q	28	15	13
H_vs_h	151	92	59	R_vs_r	22	13	9
I_vs_i	26	18	8	S_vs_s	62	22	40
J_vs_j	88	30	58	T_vs_t	24	13	11

Note: in variety code, capital letters indicate the drought group, and lowercase letters indicate the control group. Sig-up: significantly differentially expressed genes of up-regulation; Sig-down: significantly differentially expressed genes of down-regulation.

**Table 2:** MYB family KEGG annotation classification statistical table.

First Category	Second Category	Path ID	Describe	Gene ID	Varieties
Metabolism	Carbohydrate metabolism	map00040	Pentose and glucuronate interconversions	Lus.scaffold440.7	A, D, E, L, T
Organismal systems	Environmental adaptation	map04712	Circadian rhythm-plants	Lus.scaffold111.105	B, K, L, M
				Lus.scaffold292.3	B, K, M
				Lus.scaffold97.69	B, K
				Lus.scaffold80.178	K
Environmental information processing	Signal transduction	map04075	Plant hormone signal transduction	Lus.scaffold70.63	B
				Lus.scaffold45.334	B
				Lus.scaffold34.70	B

Note: the “Varieties” column shows flax varieties with differential expression of this gene. The green background indicates annotated KEGG information related to ‘Metabolism’. The orange background indicates annotated KEGG information related to ‘Organismal systems’. The blue background indicates annotated KEGG information related to ‘Environmental information processing’.

**Table 3:** GO annotation classification of the MYB family DEGs of different varieties.

GO Type	GO Term	D	L	T	A	E	K	B	M
Biological_process	Biological regulation	7	3	7	17	24	14	48	28
	Cellular process		1	4	6	17	5	26	15
	Response to stimulus	1	2	2	3	7	5	14	8
	Metabolic process	1	2	4	3	5	1	6	3
	Developmental process			2	5	8	5	8	6
	Cellular component organization or biogenesis				1	4	1	7	2
	Reproductive process				1	2	1	3	1
	Reproduction							2	
	Growth				2	2	1	3	2
	Localization							1	1
Cellular_component	Cell part	43	27	24	62	100	52	191	109
	Organelle	42	26	23	61	98	52	189	108
	Membrane				1			2	1
	Membrane part				1			7	4
	Organelle part					1		6	
	Protein-containing complex					1		5	
	Extracellular region					1		1	1
Molecular_function	Binding	26	11	14	36	55	33	113	61
	Transcription Regulator Activity	9	2	5	10	14	13	34	21
	Catalytic activity	1	2	2	3	3		9	3

Note: The numbers in the table represent the number of MYB DEGs annotated to the GO term. The yellow background represents the flax varieties belonging to ‘Strategy 1’, the green background represents the flax variety belonging to ‘Strategy 2’, the orange background represents the flax varieties belonging to ‘Strategy 3’, and the blue background represents the flax varieties belonging to ‘Strategy 4’.

**Table 4:** Names and codes of the flax varieties.

Variety Code	Variety Name	Variety Code	Variety Name	Variety Code	Variety Name	Variety Code	Variety Name
A *	Longza No.1	F	Dingya No.15	K	Lixian	P	Baya No.15
B	Zhangya No.1	G	Zhangya No.2	L	Ningya No.17	Q	Gao Lan Bai
C	Ningya No.15	H	Dingxi No.17	M	Ningya No.19	R	Longya No.10
D	Longya No.8	I	BGOLDXREDWING44X3	N	Daxuan No.3	S	Yiya No.5
E	Yiya No.4	J	R43	O	Sha Chen Zao Shu Zhong Hong	T *	Neiya No.9

Note: * indicates that the variety is water-sensitive.

## Data Availability

The transcriptome (RNA-seq) data used in this study were deposited in the Sequence Read Achieve (SRA) of the NCBI database (BioProject ID: PRJNA1046211).

## Supplementary Materials

The following supporting information can be downloaded at https://www.mdpi.com/article/10.3390/plants13050710/s1, Supplementary Table S1: Gene ID and domain of flax MYB family members; Supplementary Table S2: Statistical table of sequencing quality of flax varieties; Supplementary Table S3 Annotated MYB gene statistics for each GO term; Supplementary Table S4 Basic information of flax varieties; Supplementary Table S5: Comparison results of ‘Strategy 4’ preference genes and *Arabidopsis thaliana* MYB family homologous genes using the Blastp algorithm; Supplementary Table S6: Primer sequences used in the q-PCR experiment.
