# Supplementary material for: Analyzing the Diversity of MYB Family Response Strategies to Drought Stress in Different Flax Varieties Based on Transcriptome Data
Source: Plants (Basel). 2024 Mar 2;13(5):710. doi: 10.3390/plants13050710 (PMC10935421; doi:10.3390/plants13050710)
Supplement: Supplementary file 1 [file plants-13-00710-s001.zip › plants-2770204-supplementary.pdf]

Supplementary Table S1 Gene ID and domain of flax MYB family members

Subfamily	Gene ID	Alignment start	Alignment end	Hmm length	Conditional E-value
5R-MYB	*Lus.scaffold127.115*	331	419	89	7.60E-06
5R-MYB	*Lus.scaffold127.115*	430	472	46	7.90E-10
5R-MYB	*Lus.scaffold127.115*	481	524	46	8.90E-14
5R-MYB	*Lus.scaffold127.115*	531	575	45	3.30E-14
5R-MYB	*Lus.scaffold127.115*	583	627	45	2.20E-18
4R-MYB	*Lus.scaffold13.15*	433	475	46	8.50E-09
4R-MYB	*Lus.scaffold13.15*	484	527	46	1.60E-12
4R-MYB	*Lus.scaffold13.15*	534	578	45	7.20E-15
4R-MYB	*Lus.scaffold13.15*	586	630	45	2.70E-17
3R-MYB	*Lus.scaffold4.136*	79	125	46	8.60E-18
3R-MYB	*Lus.scaffold4.136*	131	177	46	2.20E-19
3R-MYB	*Lus.scaffold4.136*	183	226	45	6.30E-19
3R-MYB	*Lus.scaffold10.244*	37	83	46	3.20E-16
3R-MYB	*Lus.scaffold10.244*	89	135	46	5.00E-20
3R-MYB	*Lus.scaffold10.244*	141	183	44	1.00E-15
3R-MYB	*Lus.scaffold5.149*	37	83	46	1.20E-16
3R-MYB	*Lus.scaffold5.149*	89	135	46	2.10E-20
3R-MYB	*Lus.scaffold5.149*	141	183	44	1.10E-14
2R-MYB (R2R3-MYB)	*Lus.scaffold104.58*	92	138	46	2.10E-20
2R-MYB (R2R3-MYB)	*Lus.scaffold104.58*	144	186	44	1.10E-14
2R-MYB (R2R3-MYB)	*Lus.scaffold64.262*	11	56	46	2.40E-18
2R-MYB (R2R3-MYB)	*Lus.scaffold64.262*	65	107	45	1.50E-17
2R-MYB (R2R3-MYB)	*Lus.scaffold105.40*	11	56	46	3.30E-18
2R-MYB (R2R3-MYB)	*Lus.scaffold105.40*	65	107	45	4.20E-17
2R-MYB (R2R3-MYB)	*Lus.scaffold74.170*	11	56	46	3.30E-18
2R-MYB (R2R3-MYB)	*Lus.scaffold74.170*	65	107	45	4.30E-17
2R-MYB (R2R3-MYB)	*Lus.scaffold101.16*	11	56	46	2.40E-18
2R-MYB (R2R3-MYB)	*Lus.scaffold101.16*	65	107	45	6.90E-17
2R-MYB (R2R3-MYB)	*Lus.scaffold50.74*	8	55	47	4.90E-18
2R-MYB (R2R3-MYB)	*Lus.scaffold50.74*	61	105	45	2.00E-17
2R-MYB (R2R3-MYB)	*Lus.scaffold13.43*	22	69	47	1.70E-19
2R-MYB (R2R3-MYB)	*Lus.scaffold13.43*	75	118	45	2.10E-16
2R-MYB (R2R3-MYB)	*Lus.scaffold239.42*	7	52	46	1.60E-18
2R-MYB (R2R3-MYB)	*Lus.scaffold239.42*	61	103	45	8.20E-17
2R-MYB (R2R3-MYB)	*Lus.scaffold86.85*	12	57	46	9.70E-19
2R-MYB (R2R3-MYB)	*Lus.scaffold86.85*	66	108	45	2.10E-15
2R-MYB (R2R3-MYB)	*Lus.scaffold453.2*	14	61	47	1.60E-16
2R-MYB (R2R3-MYB)	*Lus.scaffold453.2*	67	111	45	1.50E-17
2R-MYB (R2R3-MYB)	*Lus.scaffold35.131*	39	84	46	9.10E-18
2R-MYB (R2R3-MYB)	*Lus.scaffold35.131*	93	135	45	9.90E-16
2R-MYB (R2R3-MYB)	*Lus.scaffold15.216*	13	58	46	1.80E-17
2R-MYB (R2R3-MYB)	*Lus.scaffold15.216*	67	109	45	7.30E-16
2R-MYB (R2R3-MYB)	*Lus.scaffold25.217*	14	61	47	3.90E-17
2R-MYB (R2R3-MYB)	*Lus.scaffold25.217*	67	112	45	4.00E-16
2R-MYB (R2R3-MYB)	*Lus.scaffold41.80*	107	153	46	2.70E-18
2R-MYB (R2R3-MYB)	*Lus.scaffold41.80*	161	201	45	5.20E-17
2R-MYB (R2R3-MYB)	*Lus.scaffold205.58*	38	83	46	2.60E-17
2R-MYB (R2R3-MYB)	*Lus.scaffold205.58*	90	133	45	6.20E-17
2R-MYB (R2R3-MYB)	*Lus.scaffold338.11*	14	59	46	2.20E-18
2R-MYB (R2R3-MYB)	*Lus.scaffold338.11*	68	110	45	2.60E-15
2R-MYB (R2R3-MYB)	*Lus.scaffold0.146*	14	61	47	1.20E-16
2R-MYB (R2R3-MYB)	*Lus.scaffold0.146*	67	112	45	1.60E-16
2R-MYB (R2R3-MYB)	*Lus.scaffold127.44*	4	50	46	1.40E-17
2R-MYB (R2R3-MYB)	*Lus.scaffold127.44*	56	99	45	1.00E-15
2R-MYB (R2R3-MYB)	*Lus.scaffold106.67*	14	61	47	6.10E-17
2R-MYB (R2R3-MYB)	*Lus.scaffold106.67*	67	112	45	5.50E-16
2R-MYB (R2R3-MYB)	*Lus.scaffold151.90*	14	61	47	1.60E-16
2R-MYB (R2R3-MYB)	*Lus.scaffold151.90*	67	111	45	6.90E-17

2R-MYB (R2R3-MYB)	*Lus.scaffold0.682*	14	61	47	1.10E-16
2R-MYB (R2R3-MYB)	*Lus.scaffold0.682*	67	112	45	1.80E-16
2R-MYB (R2R3-MYB)	*Lus.scaffold37.48*	14	61	47	6.30E-16
2R-MYB (R2R3-MYB)	*Lus.scaffold37.48*	67	111	45	2.90E-17
2R-MYB (R2R3-MYB)	*Lus.scaffold17.93*	14	61	47	1.80E-17
2R-MYB (R2R3-MYB)	*Lus.scaffold17.93*	67	110	45	9.90E-16
2R-MYB (R2R3-MYB)	*Lus.scaffold32.194*	17	64	47	2.20E-16
2R-MYB (R2R3-MYB)	*Lus.scaffold32.194*	70	113	45	6.70E-17
2R-MYB (R2R3-MYB)	*Lus.scaffold232.59*	14	61	47	1.70E-16
2R-MYB (R2R3-MYB)	*Lus.scaffold232.59*	67	111	45	1.60E-16
2R-MYB (R2R3-MYB)	*Lus.scaffold32.368*	26	73	47	5.30E-18
2R-MYB (R2R3-MYB)	*Lus.scaffold32.368*	79	123	45	1.80E-15
2R-MYB (R2R3-MYB)	*Lus.scaffold28.14*	14	61	47	7.40E-16
2R-MYB (R2R3-MYB)	*Lus.scaffold28.14*	67	112	45	3.00E-17
2R-MYB (R2R3-MYB)	*Lus.scaffold13.84*	4	50	46	1.80E-17
2R-MYB (R2R3-MYB)	*Lus.scaffold13.84*	56	99	45	1.30E-15
2R-MYB (R2R3-MYB)	*Lus.scaffold3.401*	14	61	47	2.40E-16
2R-MYB (R2R3-MYB)	*Lus.scaffold3.401*	67	112	45	4.10E-16
2R-MYB (R2R3-MYB)	*Lus.scaffold11.72*	14	61	47	7.40E-17
2R-MYB (R2R3-MYB)	*Lus.scaffold11.72*	67	110	45	2.70E-16
2R-MYB (R2R3-MYB)	*Lus.scaffold11.182*	38	83	46	3.70E-17
2R-MYB (R2R3-MYB)	*Lus.scaffold11.182*	92	134	45	1.90E-15
2R-MYB (R2R3-MYB)	*Lus.scaffold76.199*	13	58	46	3.20E-17
2R-MYB (R2R3-MYB)	*Lus.scaffold76.199*	67	109	45	6.40E-15
2R-MYB (R2R3-MYB)	*Lus.scaffold45.63*	45	91	46	2.30E-18
2R-MYB (R2R3-MYB)	*Lus.scaffold45.63*	99	139	45	7.60E-16
2R-MYB (R2R3-MYB)	*Lus.scaffold2.524*	65	110	46	5.80E-16
2R-MYB (R2R3-MYB)	*Lus.scaffold2.524*	117	160	45	2.90E-16
2R-MYB (R2R3-MYB)	*Lus.scaffold223.23*	16	63	47	5.20E-16
2R-MYB (R2R3-MYB)	*Lus.scaffold223.23*	69	113	45	1.20E-15
2R-MYB (R2R3-MYB)	*Lus.scaffold8.30*	64	109	46	5.80E-16
2R-MYB (R2R3-MYB)	*Lus.scaffold8.30*	116	159	45	2.90E-16
2R-MYB (R2R3-MYB)	*Lus.scaffold248.4*	14	61	47	4.10E-17
2R-MYB (R2R3-MYB)	*Lus.scaffold248.4*	67	112	45	1.90E-15
2R-MYB (R2R3-MYB)	*Lus.scaffold42.148*	67	114	47	1.80E-16
2R-MYB (R2R3-MYB)	*Lus.scaffold42.148*	120	163	44	2.90E-16
2R-MYB (R2R3-MYB)	*Lus.scaffold9.79*	14	61	47	5.10E-17
2R-MYB (R2R3-MYB)	*Lus.scaffold9.79*	67	112	45	2.00E-15
2R-MYB (R2R3-MYB)	*Lus.scaffold101.47*	14	61	47	5.40E-16
2R-MYB (R2R3-MYB)	*Lus.scaffold101.47*	67	111	45	2.50E-16
2R-MYB (R2R3-MYB)	*Lus.scaffold136.97*	16	63	47	2.00E-16
2R-MYB (R2R3-MYB)	*Lus.scaffold136.97*	69	113	45	7.40E-16
2R-MYB (R2R3-MYB)	*Lus.scaffold59.139*	9	56	47	1.30E-15
2R-MYB (R2R3-MYB)	*Lus.scaffold59.139*	62	107	45	7.20E-17
2R-MYB (R2R3-MYB)	*Lus.scaffold18.73*	30	77	47	1.00E-15
2R-MYB (R2R3-MYB)	*Lus.scaffold18.73*	83	126	44	2.10E-16
2R-MYB (R2R3-MYB)	*Lus.scaffold287.22*	539	586	47	9.50E-16
2R-MYB (R2R3-MYB)	*Lus.scaffold287.22*	592	635	44	1.10E-16
2R-MYB (R2R3-MYB)	*Lus.scaffold64.235*	14	61	47	7.40E-16
2R-MYB (R2R3-MYB)	*Lus.scaffold64.235*	67	111	45	2.40E-16
2R-MYB (R2R3-MYB)	*Lus.scaffold75.149*	12	57	46	1.80E-17
2R-MYB (R2R3-MYB)	*Lus.scaffold75.149*	66	108	45	5.40E-15
2R-MYB (R2R3-MYB)	*Lus.scaffold256.46*	540	587	47	9.70E-16
2R-MYB (R2R3-MYB)	*Lus.scaffold256.46*	593	636	44	1.10E-16
2R-MYB (R2R3-MYB)	*Lus.scaffold92.116*	30	77	47	1.00E-15
2R-MYB (R2R3-MYB)	*Lus.scaffold92.116*	83	126	44	3.70E-16
2R-MYB (R2R3-MYB)	*Lus.scaffold3.27*	199	245	46	1.30E-17
2R-MYB (R2R3-MYB)	*Lus.scaffold3.27*	252	293	45	1.40E-16
2R-MYB (R2R3-MYB)	*Lus.scaffold11.366*	59	106	47	2.00E-16
2R-MYB (R2R3-MYB)	*Lus.scaffold11.366*	112	155	44	6.80E-16
2R-MYB (R2R3-MYB)	*Lus.scaffold118.150*	15	62	47	1.60E-16

2R-MYB (R2R3-MYB)	*Lus.scaffold118.150*	68	112	45	1.70E-15
2R-MYB (R2R3-MYB)	*Lus.scaffold44.125*	14	61	47	7.60E-17
2R-MYB (R2R3-MYB)	*Lus.scaffold44.125*	67	112	45	2.80E-15
2R-MYB (R2R3-MYB)	*Lus.scaffold72.161*	134	180	46	1.40E-17
2R-MYB (R2R3-MYB)	*Lus.scaffold72.161*	187	228	45	1.30E-15
2R-MYB (R2R3-MYB)	*Lus.scaffold149.120*	25	72	47	1.60E-18
2R-MYB (R2R3-MYB)	*Lus.scaffold149.120*	78	122	45	1.30E-15
2R-MYB (R2R3-MYB)	*Lus.scaffold77.145*	60	105	46	2.30E-15
2R-MYB (R2R3-MYB)	*Lus.scaffold77.145*	115	155	45	1.90E-16
2R-MYB (R2R3-MYB)	*Lus.scaffold69.149*	14	61	47	1.10E-15
2R-MYB (R2R3-MYB)	*Lus.scaffold69.149*	67	111	45	3.50E-16
2R-MYB (R2R3-MYB)	*Lus.scaffold147.18*	9	56	47	1.40E-15
2R-MYB (R2R3-MYB)	*Lus.scaffold147.18*	62	107	45	1.90E-16
2R-MYB (R2R3-MYB)	*Lus.scaffold193.11*	181	227	46	1.70E-13
2R-MYB (R2R3-MYB)	*Lus.scaffold193.11*	233	275	45	2.20E-19
2R-MYB (R2R3-MYB)	*Lus.scaffold59.140*	14	61	47	3.50E-14
2R-MYB (R2R3-MYB)	*Lus.scaffold59.140*	67	112	45	5.90E-18
2R-MYB (R2R3-MYB)	*Lus.scaffold19.170*	14	61	47	3.10E-17
2R-MYB (R2R3-MYB)	*Lus.scaffold19.170*	69	110	45	2.60E-14
2R-MYB (R2R3-MYB)	*Lus.scaffold159.64*	14	61	47	7.00E-16
2R-MYB (R2R3-MYB)	*Lus.scaffold159.64*	67	111	45	5.60E-16
2R-MYB (R2R3-MYB)	*Lus.scaffold191.31*	14	61	47	5.00E-16
2R-MYB (R2R3-MYB)	*Lus.scaffold191.31*	67	112	45	1.20E-16
2R-MYB (R2R3-MYB)	*Lus.scaffold32.297*	14	61	47	2.90E-16
2R-MYB (R2R3-MYB)	*Lus.scaffold32.297*	67	112	45	2.40E-15
2R-MYB (R2R3-MYB)	*Lus.scaffold20.89*	14	61	47	1.60E-16
2R-MYB (R2R3-MYB)	*Lus.scaffold20.89*	67	109	44	8.10E-15
2R-MYB (R2R3-MYB)	*Lus.scaffold188.9*	14	61	47	2.40E-16
2R-MYB (R2R3-MYB)	*Lus.scaffold188.9*	67	111	45	1.80E-15
2R-MYB (R2R3-MYB)	*Lus.scaffold217.66*	14	61	47	2.40E-16
2R-MYB (R2R3-MYB)	*Lus.scaffold217.66*	67	111	45	1.80E-15
2R-MYB (R2R3-MYB)	*Lus.scaffold30.134*	14	61	47	3.00E-17
2R-MYB (R2R3-MYB)	*Lus.scaffold30.134*	69	110	45	4.30E-14
2R-MYB (R2R3-MYB)	*Lus.scaffold17.356*	46	91	46	2.00E-17
2R-MYB (R2R3-MYB)	*Lus.scaffold17.356*	98	141	45	1.00E-15
2R-MYB (R2R3-MYB)	*Lus.scaffold231.37*	14	61	47	4.30E-16
2R-MYB (R2R3-MYB)	*Lus.scaffold231.37*	67	112	45	4.30E-15
2R-MYB (R2R3-MYB)	*Lus.scaffold259.57*	14	61	47	6.20E-17
2R-MYB (R2R3-MYB)	*Lus.scaffold259.57*	67	112	45	1.90E-14
2R-MYB (R2R3-MYB)	*Lus.scaffold150.25*	22	69	47	1.00E-15
2R-MYB (R2R3-MYB)	*Lus.scaffold150.25*	75	118	45	6.30E-16
2R-MYB (R2R3-MYB)	*Lus.scaffold88.20*	20	67	47	1.00E-15
2R-MYB (R2R3-MYB)	*Lus.scaffold88.20*	73	116	45	6.40E-16
2R-MYB (R2R3-MYB)	*Lus.scaffold378.23*	112	158	46	9.80E-17
2R-MYB (R2R3-MYB)	*Lus.scaffold378.23*	165	206	45	1.20E-15
2R-MYB (R2R3-MYB)	*Lus.scaffold30.88*	14	61	47	1.10E-16
2R-MYB (R2R3-MYB)	*Lus.scaffold30.88*	67	112	45	2.20E-14
2R-MYB (R2R3-MYB)	*Lus.scaffold63.283*	32	79	47	2.60E-16
2R-MYB (R2R3-MYB)	*Lus.scaffold63.283*	85	128	45	6.40E-16
2R-MYB (R2R3-MYB)	*Lus.scaffold39.293*	14	61	47	8.90E-17
2R-MYB (R2R3-MYB)	*Lus.scaffold39.293*	67	112	45	1.20E-14
2R-MYB (R2R3-MYB)	*Lus.scaffold102.77*	59	104	46	2.30E-15
2R-MYB (R2R3-MYB)	*Lus.scaffold102.77*	114	154	45	6.20E-16
2R-MYB (R2R3-MYB)	*Lus.scaffold312.10*	116	162	46	4.40E-16
2R-MYB (R2R3-MYB)	*Lus.scaffold312.10*	168	210	44	3.80E-17
2R-MYB (R2R3-MYB)	*Lus.scaffold70.198*	73	118	46	8.50E-16
2R-MYB (R2R3-MYB)	*Lus.scaffold70.198*	125	168	45	1.50E-15
2R-MYB (R2R3-MYB)	*Lus.scaffold96.15*	14	61	47	4.40E-16
2R-MYB (R2R3-MYB)	*Lus.scaffold96.15*	67	112	45	9.50E-15
2R-MYB (R2R3-MYB)	*Lus.scaffold147.17*	14	61	47	1.20E-14
2R-MYB (R2R3-MYB)	*Lus.scaffold147.17*	67	112	45	2.50E-17

2R-MYB (R2R3-MYB)	*Lus.scaffold102.115*	14	61	47	2.20E-16
2R-MYB (R2R3-MYB)	*Lus.scaffold102.115*	67	112	45	2.20E-14
2R-MYB (R2R3-MYB)	*Lus.scaffold77.112*	14	61	47	2.10E-16
2R-MYB (R2R3-MYB)	*Lus.scaffold77.112*	67	112	45	2.20E-14
2R-MYB (R2R3-MYB)	*Lus.scaffold0.141*	14	61	47	7.80E-17
2R-MYB (R2R3-MYB)	*Lus.scaffold0.141*	67	112	45	3.00E-14
2R-MYB (R2R3-MYB)	*Lus.scaffold69.176*	14	61	47	5.60E-14
2R-MYB (R2R3-MYB)	*Lus.scaffold69.176*	67	112	45	4.50E-17
2R-MYB (R2R3-MYB)	*Lus.scaffold51.187*	14	61	47	8.00E-17
2R-MYB (R2R3-MYB)	*Lus.scaffold51.187*	67	110	45	3.10E-14
2R-MYB (R2R3-MYB)	*Lus.scaffold18.313*	14	61	47	8.00E-17
2R-MYB (R2R3-MYB)	*Lus.scaffold18.313*	67	110	45	3.10E-14
2R-MYB (R2R3-MYB)	*Lus.scaffold196.102*	21	68	47	1.60E-14
2R-MYB (R2R3-MYB)	*Lus.scaffold196.102*	74	117	45	1.00E-15
2R-MYB (R2R3-MYB)	*Lus.scaffold2.413*	18	65	47	2.20E-15
2R-MYB (R2R3-MYB)	*Lus.scaffold2.413*	71	115	45	5.60E-15
2R-MYB (R2R3-MYB)	*Lus.scaffold8.155*	18	65	47	2.30E-15
2R-MYB (R2R3-MYB)	*Lus.scaffold8.155*	71	115	45	5.70E-15
2R-MYB (R2R3-MYB)	*Lus.scaffold133.21*	9	56	47	3.40E-16
2R-MYB (R2R3-MYB)	*Lus.scaffold133.21*	62	106	45	1.00E-14
2R-MYB (R2R3-MYB)	*Lus.scaffold83.114*	14	61	47	3.40E-16
2R-MYB (R2R3-MYB)	*Lus.scaffold83.114*	67	111	45	1.00E-14
2R-MYB (R2R3-MYB)	*Lus.scaffold326.2*	14	61	47	2.70E-16
2R-MYB (R2R3-MYB)	*Lus.scaffold326.2*	67	112	45	6.00E-14
2R-MYB (R2R3-MYB)	*Lus.scaffold30.79*	14	61	47	5.60E-15
2R-MYB (R2R3-MYB)	*Lus.scaffold30.79*	67	112	45	1.20E-16
2R-MYB (R2R3-MYB)	*Lus.scaffold80.38*	14	61	47	3.70E-15
2R-MYB (R2R3-MYB)	*Lus.scaffold80.38*	67	112	45	3.40E-15
2R-MYB (R2R3-MYB)	*Lus.scaffold21.181*	183	229	46	1.00E-16
2R-MYB (R2R3-MYB)	*Lus.scaffold21.181*	235	276	45	2.80E-15
2R-MYB (R2R3-MYB)	*Lus.scaffold203.26*	5	50	47	6.30E-17
2R-MYB (R2R3-MYB)	*Lus.scaffold203.26*	56	98	45	5.40E-14
2R-MYB (R2R3-MYB)	*Lus.scaffold279.32*	14	61	47	3.80E-15
2R-MYB (R2R3-MYB)	*Lus.scaffold279.32*	67	112	45	2.50E-15
2R-MYB (R2R3-MYB)	*Lus.scaffold75.205*	14	61	47	1.40E-16
2R-MYB (R2R3-MYB)	*Lus.scaffold75.205*	67	112	45	2.20E-13
2R-MYB (R2R3-MYB)	*Lus.scaffold8.490*	31	78	47	1.50E-15
2R-MYB (R2R3-MYB)	*Lus.scaffold8.490*	84	127	45	2.30E-15
2R-MYB (R2R3-MYB)	*Lus.scaffold13.232*	16	63	47	6.90E-15
2R-MYB (R2R3-MYB)	*Lus.scaffold13.232*	69	114	45	4.30E-15
2R-MYB (R2R3-MYB)	*Lus.scaffold55.245*	17	61	47	8.30E-16
2R-MYB (R2R3-MYB)	*Lus.scaffold55.245*	67	109	44	3.40E-14
2R-MYB (R2R3-MYB)	*Lus.scaffold133.113*	14	61	47	1.10E-14
2R-MYB (R2R3-MYB)	*Lus.scaffold133.113*	67	112	45	1.30E-15
2R-MYB (R2R3-MYB)	*Lus.scaffold13.131*	21	68	47	3.30E-14
2R-MYB (R2R3-MYB)	*Lus.scaffold13.131*	74	117	45	1.10E-15
2R-MYB (R2R3-MYB)	*Lus.scaffold45.213*	44	91	47	1.50E-15
2R-MYB (R2R3-MYB)	*Lus.scaffold45.213*	97	140	45	2.30E-15
2R-MYB (R2R3-MYB)	*Lus.scaffold35.141*	14	61	47	6.90E-16
2R-MYB (R2R3-MYB)	*Lus.scaffold35.141*	67	111	45	1.50E-14
2R-MYB (R2R3-MYB)	*Lus.scaffold34.195*	78	123	46	8.90E-16
2R-MYB (R2R3-MYB)	*Lus.scaffold34.195*	130	173	45	1.60E-15
2R-MYB (R2R3-MYB)	*Lus.scaffold61.54*	15	62	47	7.40E-17
2R-MYB (R2R3-MYB)	*Lus.scaffold61.54*	70	111	45	5.00E-13
2R-MYB (R2R3-MYB)	*Lus.scaffold31.283*	29	76	47	2.50E-16
2R-MYB (R2R3-MYB)	*Lus.scaffold31.283*	82	125	45	1.40E-14
2R-MYB (R2R3-MYB)	*Lus.scaffold0.369*	14	61	47	9.60E-16
2R-MYB (R2R3-MYB)	*Lus.scaffold0.369*	67	112	45	3.20E-14
2R-MYB (R2R3-MYB)	*Lus.scaffold83.193*	14	61	47	1.10E-14
2R-MYB (R2R3-MYB)	*Lus.scaffold83.193*	67	112	45	3.50E-15
2R-MYB (R2R3-MYB)	*Lus.scaffold147.130*	15	62	47	4.60E-16

2R-MYB (R2R3-MYB)	*Lus.scaffold147.130*	68	112	45	5.10E-14
2R-MYB (R2R3-MYB)	*Lus.scaffold81.169*	15	62	47	7.60E-17
2R-MYB (R2R3-MYB)	*Lus.scaffold81.169*	70	111	45	5.10E-13
2R-MYB (R2R3-MYB)	*Lus.scaffold137.65*	16	63	47	1.10E-14
2R-MYB (R2R3-MYB)	*Lus.scaffold137.65*	69	114	45	4.30E-15
2R-MYB (R2R3-MYB)	*Lus.scaffold133.20*	16	63	47	8.70E-17
2R-MYB (R2R3-MYB)	*Lus.scaffold133.20*	70	113	45	2.80E-13
2R-MYB (R2R3-MYB)	*Lus.scaffold101.180*	13	60	47	1.40E-14
2R-MYB (R2R3-MYB)	*Lus.scaffold101.180*	70	111	45	4.50E-16
2R-MYB (R2R3-MYB)	*Lus.scaffold354.6*	21	68	47	1.40E-14
2R-MYB (R2R3-MYB)	*Lus.scaffold354.6*	74	117	45	9.30E-15
2R-MYB (R2R3-MYB)	*Lus.scaffold64.107*	11	58	47	2.80E-14
2R-MYB (R2R3-MYB)	*Lus.scaffold64.107*	68	109	43	5.10E-16
2R-MYB (R2R3-MYB)	*Lus.scaffold39.294*	14	61	47	5.70E-14
2R-MYB (R2R3-MYB)	*Lus.scaffold39.294*	67	112	45	9.70E-16
2R-MYB (R2R3-MYB)	*Lus.scaffold83.113*	9	56	47	1.30E-16
2R-MYB (R2R3-MYB)	*Lus.scaffold83.113*	63	106	45	3.20E-13
2R-MYB (R2R3-MYB)	*Lus.scaffold69.175*	14	61	47	5.90E-16
2R-MYB (R2R3-MYB)	*Lus.scaffold69.175*	67	112	45	1.20E-13
2R-MYB (R2R3-MYB)	*Lus.scaffold13.394*	14	61	47	2.30E-14
2R-MYB (R2R3-MYB)	*Lus.scaffold13.394*	67	112	45	1.00E-14
2R-MYB (R2R3-MYB)	*Lus.scaffold43.301*	23	70	47	7.70E-14
2R-MYB (R2R3-MYB)	*Lus.scaffold43.301*	76	121	45	2.90E-15
2R-MYB (R2R3-MYB)	*Lus.scaffold83.58*	14	61	47	6.60E-14
2R-MYB (R2R3-MYB)	*Lus.scaffold83.58*	71	111	45	2.00E-15
2R-MYB (R2R3-MYB)	*Lus.scaffold199.77*	14	61	47	6.80E-14
2R-MYB (R2R3-MYB)	*Lus.scaffold199.77*	71	111	45	2.10E-15
2R-MYB (R2R3-MYB)	*Lus.scaffold24.203*	29	73	45	1.70E-14
2R-MYB (R2R3-MYB)	*Lus.scaffold24.203*	79	122	44	9.20E-15
2R-MYB (R2R3-MYB)	*Lus.scaffold10.29*	14	61	47	2.10E-15
2R-MYB (R2R3-MYB)	*Lus.scaffold10.29*	67	111	45	9.10E-14
2R-MYB (R2R3-MYB)	*Lus.scaffold46.280*	14	61	47	2.10E-15
2R-MYB (R2R3-MYB)	*Lus.scaffold46.280*	67	111	45	9.30E-14
2R-MYB (R2R3-MYB)	*Lus.scaffold66.39*	29	73	45	1.30E-14
2R-MYB (R2R3-MYB)	*Lus.scaffold66.39*	79	122	44	2.50E-14
2R-MYB (R2R3-MYB)	*Lus.scaffold68.86*	14	61	47	2.30E-14
2R-MYB (R2R3-MYB)	*Lus.scaffold68.86*	67	112	45	2.40E-14
2R-MYB (R2R3-MYB)	*Lus.scaffold39.171*	43	90	47	2.80E-14
2R-MYB (R2R3-MYB)	*Lus.scaffold39.171*	96	139	45	1.00E-14
2R-MYB (R2R3-MYB)	*Lus.scaffold219.72*	22	69	47	1.30E-13
2R-MYB (R2R3-MYB)	*Lus.scaffold219.72*	75	119	45	4.80E-15
2R-MYB (R2R3-MYB)	*Lus.scaffold110.62*	14	61	47	1.80E-15
2R-MYB (R2R3-MYB)	*Lus.scaffold110.62*	72	111	45	8.50E-14
2R-MYB (R2R3-MYB)	*Lus.scaffold223.21*	14	61	47	3.70E-14
2R-MYB (R2R3-MYB)	*Lus.scaffold223.21*	68	111	45	2.80E-15
2R-MYB (R2R3-MYB)	*Lus.scaffold69.174*	14	61	47	5.20E-15
2R-MYB (R2R3-MYB)	*Lus.scaffold69.174*	67	112	45	6.90E-14
2R-MYB (R2R3-MYB)	*Lus.scaffold31.241*	33	79	47	9.30E-15
2R-MYB (R2R3-MYB)	*Lus.scaffold31.241*	86	129	45	6.10E-14
2R-MYB (R2R3-MYB)	*Lus.scaffold846.1*	21	68	47	6.80E-14
2R-MYB (R2R3-MYB)	*Lus.scaffold846.1*	74	119	45	8.20E-15
2R-MYB (R2R3-MYB)	*Lus.scaffold197.90*	21	68	47	7.70E-14
2R-MYB (R2R3-MYB)	*Lus.scaffold197.90*	74	119	45	9.30E-15
2R-MYB (R2R3-MYB)	*Lus.scaffold6.31*	11	58	47	1.90E-14
2R-MYB (R2R3-MYB)	*Lus.scaffold6.31*	64	109	45	4.70E-14
2R-MYB (R2R3-MYB)	*Lus.scaffold54.116*	12	59	47	2.00E-14
2R-MYB (R2R3-MYB)	*Lus.scaffold54.116*	65	110	45	4.80E-14
2R-MYB (R2R3-MYB)	*Lus.scaffold46.328*	28	75	47	8.00E-15
2R-MYB (R2R3-MYB)	*Lus.scaffold46.328*	81	124	45	5.30E-14
2R-MYB (R2R3-MYB)	*Lus.scaffold70.240*	35	79	45	1.40E-13
2R-MYB (R2R3-MYB)	*Lus.scaffold70.240*	85	128	44	1.70E-14

2R-MYB (R2R3-MYB)	*Lus.scaffold34.229*	33	77	45	1.40E-13
2R-MYB (R2R3-MYB)	*Lus.scaffold34.229*	83	126	44	1.70E-14
2R-MYB (R2R3-MYB)	*Lus.scaffold69.1*	37	84	47	1.60E-13
2R-MYB (R2R3-MYB)	*Lus.scaffold69.1*	90	133	45	2.30E-14
2R-MYB (R2R3-MYB)	*Lus.scaffold71.104*	38	85	47	1.60E-13
2R-MYB (R2R3-MYB)	*Lus.scaffold71.104*	91	134	45	2.30E-14
2R-MYB (R2R3-MYB)	*Lus.scaffold150.40*	14	61	47	1.50E-12
2R-MYB (R2R3-MYB)	*Lus.scaffold150.40*	67	110	45	9.10E-16
2R-MYB (R2R3-MYB)	*Lus.scaffold53.124*	14	62	48	7.10E-16
2R-MYB (R2R3-MYB)	*Lus.scaffold53.124*	69	111	45	6.60E-12
2R-MYB (R2R3-MYB)	*Lus.scaffold69.140*	14	61	47	5.30E-15
2R-MYB (R2R3-MYB)	*Lus.scaffold69.140*	68	111	45	2.00E-13
2R-MYB (R2R3-MYB)	*Lus.scaffold88.34*	14	61	47	2.70E-12
2R-MYB (R2R3-MYB)	*Lus.scaffold88.34*	67	110	45	9.70E-16
2R-MYB (R2R3-MYB)	*Lus.scaffold165.140*	14	61	47	3.00E-15
2R-MYB (R2R3-MYB)	*Lus.scaffold165.140*	71	110	42	3.00E-13
2R-MYB (R2R3-MYB)	*Lus.scaffold45.326*	14	61	47	7.10E-13
2R-MYB (R2R3-MYB)	*Lus.scaffold45.326*	67	112	45	3.10E-15
2R-MYB (R2R3-MYB)	*Lus.scaffold265.14*	17	66	49	1.70E-12
2R-MYB (R2R3-MYB)	*Lus.scaffold265.14*	72	116	45	3.60E-15
2R-MYB (R2R3-MYB)	*Lus.scaffold68.85*	16	63	47	2.70E-13
2R-MYB (R2R3-MYB)	*Lus.scaffold68.85*	69	114	45	8.90E-15
2R-MYB (R2R3-MYB)	*Lus.scaffold8.374*	14	61	47	9.30E-13
2R-MYB (R2R3-MYB)	*Lus.scaffold8.374*	67	112	45	3.10E-15
2R-MYB (R2R3-MYB)	*Lus.scaffold290.52*	14	62	48	1.80E-15
2R-MYB (R2R3-MYB)	*Lus.scaffold290.52*	69	111	45	6.90E-12
2R-MYB (R2R3-MYB)	*Lus.scaffold155.16*	22	69	47	1.30E-11
2R-MYB (R2R3-MYB)	*Lus.scaffold155.16*	75	119	45	7.30E-16
2R-MYB (R2R3-MYB)	*Lus.scaffold36.180*	14	62	48	5.60E-16
2R-MYB (R2R3-MYB)	*Lus.scaffold36.180*	69	111	45	2.50E-11
2R-MYB (R2R3-MYB)	*Lus.scaffold157.106*	14	62	48	5.70E-16
2R-MYB (R2R3-MYB)	*Lus.scaffold157.106*	69	111	45	2.60E-11
2R-MYB (R2R3-MYB)	*Lus.scaffold16.451*	14	62	48	3.90E-15
2R-MYB (R2R3-MYB)	*Lus.scaffold16.451*	69	111	45	7.40E-12
2R-MYB (R2R3-MYB)	*Lus.scaffold13.393*	16	63	47	1.60E-12
2R-MYB (R2R3-MYB)	*Lus.scaffold13.393*	69	114	45	4.00E-15
2R-MYB (R2R3-MYB)	*Lus.scaffold29.280*	43	90	47	1.60E-12
2R-MYB (R2R3-MYB)	*Lus.scaffold29.280*	96	139	45	5.20E-15
2R-MYB (R2R3-MYB)	*Lus.scaffold109.55*	14	62	48	2.00E-15
2R-MYB (R2R3-MYB)	*Lus.scaffold109.55*	69	111	45	1.60E-11
2R-MYB (R2R3-MYB)	*Lus.scaffold83.57*	14	61	47	3.80E-12
2R-MYB (R2R3-MYB)	*Lus.scaffold83.57*	71	111	45	4.00E-15
2R-MYB (R2R3-MYB)	*Lus.scaffold206.64*	14	62	48	2.10E-15
2R-MYB (R2R3-MYB)	*Lus.scaffold206.64*	69	111	45	1.70E-11
2R-MYB (R2R3-MYB)	*Lus.scaffold20.444*	14	62	48	2.50E-15
2R-MYB (R2R3-MYB)	*Lus.scaffold20.444*	69	111	45	1.70E-11
2R-MYB (R2R3-MYB)	*Lus.scaffold353.10*	14	62	48	9.60E-15
2R-MYB (R2R3-MYB)	*Lus.scaffold353.10*	69	111	45	8.70E-12
2R-MYB (R2R3-MYB)	*Lus.scaffold152.51*	14	61	47	1.70E-12
2R-MYB (R2R3-MYB)	*Lus.scaffold152.51*	67	112	45	2.60E-14
2R-MYB (R2R3-MYB)	*Lus.scaffold440.7*	17	66	49	1.80E-12
2R-MYB (R2R3-MYB)	*Lus.scaffold440.7*	72	116	45	5.10E-14
2R-MYB (R2R3-MYB)	*Lus.scaffold223.20*	14	61	47	1.50E-13
2R-MYB (R2R3-MYB)	*Lus.scaffold223.20*	67	110	45	5.80E-14
2R-MYB (R2R3-MYB)	*Lus.scaffold131.105*	14	62	48	7.80E-15
2R-MYB (R2R3-MYB)	*Lus.scaffold131.105*	69	111	45	1.70E-11
2R-MYB (R2R3-MYB)	*Lus.scaffold39.256*	13	60	47	4.40E-14
2R-MYB (R2R3-MYB)	*Lus.scaffold39.256*	66	111	46	1.80E-13
2R-MYB (R2R3-MYB)	*Lus.scaffold43.50*	14	61	47	1.00E-14
2R-MYB (R2R3-MYB)	*Lus.scaffold43.50*	72	111	45	2.70E-12
2R-MYB (R2R3-MYB)	*Lus.scaffold0.168*	14	61	47	1.90E-14

2R-MYB (R2R3-MYB)	*Lus.scaffold0.168*	67	112	45	1.20E-14
2R-MYB (R2R3-MYB)	*Lus.scaffold136.93*	14	61	47	1.50E-13
2R-MYB (R2R3-MYB)	*Lus.scaffold136.93*	67	110	45	7.50E-14
2R-MYB (R2R3-MYB)	*Lus.scaffold68.83*	15	62	47	3.60E-12
2R-MYB (R2R3-MYB)	*Lus.scaffold68.83*	68	113	45	5.00E-14
2R-MYB (R2R3-MYB)	*Lus.scaffold13.392*	15	62	47	5.10E-13
2R-MYB (R2R3-MYB)	*Lus.scaffold13.392*	68	113	45	3.00E-13
2R-MYB (R2R3-MYB)	*Lus.scaffold46.145*	37	104	67	2.80E-09
2R-MYB (R2R3-MYB)	*Lus.scaffold46.145*	110	160	54	6.10E-16
2R-MYB (R2R3-MYB)	*Lus.scaffold69.46*	14	62	48	1.60E-15
2R-MYB (R2R3-MYB)	*Lus.scaffold69.46*	69	111	45	5.60E-10
2R-MYB (R2R3-MYB)	*Lus.scaffold13.391*	13	60	47	3.60E-12
2R-MYB (R2R3-MYB)	*Lus.scaffold13.391*	66	111	45	1.70E-13
2R-MYB (R2R3-MYB)	*Lus.scaffold0.704*	14	61	47	3.50E-13
2R-MYB (R2R3-MYB)	*Lus.scaffold0.704*	67	112	45	5.60E-14
2R-MYB (R2R3-MYB)	*Lus.scaffold80.117*	14	61	47	2.10E-14
2R-MYB (R2R3-MYB)	*Lus.scaffold80.117*	67	112	45	7.70E-11
2R-MYB (R2R3-MYB)	*Lus.scaffold73.142*	14	64	50	2.00E-10
2R-MYB (R2R3-MYB)	*Lus.scaffold73.142*	70	114	45	2.20E-14
2R-MYB (R2R3-MYB)	*Lus.scaffold0.833*	14	61	47	1.20E-11
2R-MYB (R2R3-MYB)	*Lus.scaffold0.833*	67	112	45	2.00E-13
2R-MYB (R2R3-MYB)	*Lus.scaffold33.131*	10	55	47	3.40E-13
2R-MYB (R2R3-MYB)	*Lus.scaffold33.131*	66	105	43	3.10E-12
2R-MYB (R2R3-MYB)	*Lus.scaffold198.54*	14	61	47	8.30E-13
2R-MYB (R2R3-MYB)	*Lus.scaffold198.54*	67	112	45	1.10E-11
2R-MYB (R2R3-MYB)	*Lus.scaffold100.96*	15	65	50	6.70E-11
2R-MYB (R2R3-MYB)	*Lus.scaffold100.96*	71	114	45	9.10E-13
2R-MYB (R2R3-MYB)	*Lus.scaffold156.27*	16	46	32	6.90E-10
2R-MYB (R2R3-MYB)	*Lus.scaffold156.27*	55	97	45	6.00E-16
2R-MYB (R2R3-MYB)	*Lus.scaffold122.2*	4	53	49	9.30E-14
2R-MYB (R2R3-MYB)	*Lus.scaffold122.2*	59	94	40	2.20E-10
2R-MYB (R2R3-MYB)	*Lus.scaffold185.48*	2	30	29	4.60E-09
2R-MYB (R2R3-MYB)	*Lus.scaffold185.48*	37	78	45	1.20E-14
2R-MYB (R2R3-MYB)	*Lus.scaffold4.134*	4	53	49	1.30E-13
2R-MYB (R2R3-MYB)	*Lus.scaffold4.134*	59	94	40	2.20E-10
2R-MYB (R2R3-MYB)	*Lus.scaffold48.227*	8	53	46	1.30E-12
2R-MYB (R2R3-MYB)	*Lus.scaffold48.227*	60	103	44	3.50E-10
2R-MYB (R2R3-MYB)	*Lus.scaffold82.98*	8	53	46	1.20E-12
2R-MYB (R2R3-MYB)	*Lus.scaffold82.98*	60	103	44	3.40E-10
2R-MYB (R2R3-MYB)	*Lus.scaffold65.70*	352	389	42	7.70E-07
2R-MYB (R2R3-MYB)	*Lus.scaffold65.70*	401	463	63	5.90E-07
2R-MYB (R2R3-MYB)	*Lus.scaffold272.65*	338	378	44	1.30E-06
2R-MYB (R2R3-MYB)	*Lus.scaffold272.65*	388	449	63	2.20E-07
1R-MYB(MYB-Related)	*Lus.scaffold236.34*	14	61	47	7.80E-15
1R-MYB (MYB-Related)	*Lus.scaffold236.34*	73	111	41	4.90E-14
1R-MYB (MYB-Related)	*Lus.scaffold200.70*	14	61	47	7.90E-15
1R-MYB (MYB-Related)	*Lus.scaffold200.70*	73	111	41	5.10E-14
1R-MYB (MYB-Related)	*Lus.scaffold34.186*	14	61	47	1.90E-18
1R-MYB (MYB-Related)	*Lus.scaffold117.96*	26	73	49	1.20E-08
1R-MYB (MYB-Related)	*Lus.scaffold117.96*	136	180	47	2.00E-13
1R-MYB (MYB-Related)	*Lus.scaffold169.129*	26	73	49	1.20E-08
1R-MYB (MYB-Related)	*Lus.scaffold169.129*	136	180	47	2.00E-13
1R-MYB (MYB-Related)	*Lus.scaffold45.51*	29	75	49	5.80E-07
1R-MYB (MYB-Related)	*Lus.scaffold45.51*	139	183	47	2.60E-14
1R-MYB (MYB-Related)	*Lus.scaffold75.255*	21	67	50	2.40E-08
1R-MYB (MYB-Related)	*Lus.scaffold75.255*	126	170	47	7.70E-13
1R-MYB (MYB-Related)	*Lus.scaffold41.67*	29	75	49	7.50E-07
1R-MYB (MYB-Related)	*Lus.scaffold41.67*	139	183	47	2.60E-14
1R-MYB (MYB-Related)	*Lus.scaffold17.143*	4	50	47	3.30E-18
1R-MYB (MYB-Related)	*Lus.scaffold2.510*	25	71	50	2.00E-07
1R-MYB (MYB-Related)	*Lus.scaffold2.510*	125	169	47	2.70E-13

1R-MYB (MYB-Related)	*Lus.scaffold193.10*	299	342	45	1.80E-16
1R-MYB (MYB-Related)	*Lus.scaffold132.143*	5	50	47	2.60E-18
1R-MYB (MYB-Related)	*Lus.scaffold315.32*	11	54	49	1.30E-07
1R-MYB (MYB-Related)	*Lus.scaffold315.32*	123	167	45	7.20E-13
1R-MYB (MYB-Related)	*Lus.scaffold46.61*	21	66	49	2.60E-07
1R-MYB (MYB-Related)	*Lus.scaffold46.61*	125	169	47	6.90E-13
1R-MYB (MYB-Related)	*Lus.scaffold70.188*	14	61	47	5.20E-18
1R-MYB (MYB-Related)	*Lus.scaffold69.142*	51	95	45	1.10E-14
1R-MYB (MYB-Related)	*Lus.scaffold0.296*	11	54	49	4.90E-07
1R-MYB (MYB-Related)	*Lus.scaffold0.296*	122	166	45	7.20E-13
1R-MYB (MYB-Related)	*Lus.scaffold126.85*	119	163	47	2.20E-14
1R-MYB (MYB-Related)	*Lus.scaffold8.45*	21	69	51	1.40E-07
1R-MYB (MYB-Related)	*Lus.scaffold8.45*	124	168	47	3.30E-12
1R-MYB (MYB-Related)	*Lus.scaffold152.10*	33	80	47	1.00E-13
1R-MYB (MYB-Related)	*Lus.scaffold0.747*	114	158	47	4.50E-12
1R-MYB (MYB-Related)	*Lus.scaffold146.109*	345	381	38	6.10E-07
1R-MYB (MYB-Related)	*Lus.scaffold146.109*	394	456	63	9.60E-07
1R-MYB (MYB-Related)	*Lus.scaffold84.219*	16	73	57	1.20E-13
1R-MYB (MYB-Related)	*Lus.scaffold2.688*	800	840	44	5.10E-07
1R-MYB (MYB-Related)	*Lus.scaffold2.688*	1006	1046	45	1.90E-09
1R-MYB (MYB-Related)	*Lus.scaffold259.59*	14	59	45	2.10E-13
1R-MYB (MYB-Related)	*Lus.scaffold263.11*	784	825	44	1.80E-06
1R-MYB (MYB-Related)	*Lus.scaffold263.11*	992	1031	44	4.90E-09
1R-MYB (MYB-Related)	*Lus.scaffold0.634*	789	830	44	3.60E-06
1R-MYB (MYB-Related)	*Lus.scaffold0.634*	997	1038	45	3.50E-09
1R-MYB (MYB-Related)	*Lus.scaffold0.207*	112	156	47	4.40E-12
1R-MYB (MYB-Related)	*Lus.scaffold139.125*	58	101	45	1.80E-16
1R-MYB (MYB-Related)	*Lus.scaffold96.114*	70	113	45	1.80E-16
1R-MYB (MYB-Related)	*Lus.scaffold344.22*	2	42	42	2.70E-16
1R-MYB (MYB-Related)	*Lus.scaffold13.91*	71	114	45	5.90E-16
1R-MYB (MYB-Related)	*Lus.scaffold127.37*	59	102	45	8.30E-16
1R-MYB (MYB-Related)	*Lus.scaffold144.133*	795	835	44	8.50E-07
1R-MYB (MYB-Related)	*Lus.scaffold144.133*	1001	1040	44	1.30E-07
1R-MYB (MYB-Related)	*Lus.scaffold90.185*	246	287	44	4.00E-15
1R-MYB (MYB-Related)	*Lus.scaffold393.20*	69	112	45	1.20E-14
1R-MYB (MYB-Related)	*Lus.scaffold16.256*	245	286	44	8.10E-15
1R-MYB (MYB-Related)	*Lus.scaffold292.3*	24	67	45	1.30E-14
1R-MYB (MYB-Related)	*Lus.scaffold111.105*	24	67	45	1.30E-14
1R-MYB (MYB-Related)	*Lus.scaffold97.69*	24	67	45	3.00E-14
1R-MYB (MYB-Related)	*Lus.scaffold6.348*	592	635	49	2.40E-07
1R-MYB (MYB-Related)	*Lus.scaffold80.178*	24	67	45	3.70E-14
1R-MYB (MYB-Related)	*Lus.scaffold32.197*	196	234	40	3.00E-08
1R-MYB (MYB-Related)	*Lus.scaffold168.79*	53	97	45	1.60E-13
1R-MYB (MYB-Related)	*Lus.scaffold168.80*	53	97	45	1.60E-13
1R-MYB (MYB-Related)	*Lus.scaffold183.68*	136	180	45	2.10E-13
1R-MYB (MYB-Related)	*Lus.scaffold170.74*	355	397	45	2.50E-13
1R-MYB (MYB-Related)	*Lus.scaffold381.22*	313	355	45	6.60E-13
1R-MYB (MYB-Related)	*Lus.scaffold0.771*	80	124	45	7.80E-13
1R-MYB (MYB-Related)	*Lus.scaffold335.10*	80	124	45	8.00E-13
1R-MYB (MYB-Related)	*Lus.scaffold99.140*	107	150	45	9.70E-13
1R-MYB (MYB-Related)	*Lus.scaffold130.65*	593	636	49	1.40E-07
1R-MYB (MYB-Related)	*Lus.scaffold130.64*	593	636	49	1.40E-07
1R-MYB (MYB-Related)	*Lus.scaffold6.349*	597	640	49	1.60E-07
1R-MYB (MYB-Related)	*Lus.scaffold130.63*	593	636	49	1.40E-07
1R-MYB (MYB-Related)	*Lus.scaffold15.499*	107	150	45	9.70E-13
1R-MYB (MYB-Related)	*Lus.scaffold437.3*	104	148	47	1.50E-12
1R-MYB (MYB-Related)	*Lus.scaffold687.1*	596	639	49	1.40E-07
1R-MYB (MYB-Related)	*Lus.scaffold166.26*	119	163	47	2.10E-12
1R-MYB (MYB-Related)	*Lus.scaffold2.457*	9	53	47	3.00E-12
1R-MYB (MYB-Related)	*Lus.scaffold96.147*	365	408	46	5.10E-12
1R-MYB (MYB-Related)	*Lus.scaffold8.183*	5	56	52	3.60E-12

1R-MYB (MYB-Related)	*Lus.scaffold55.180*	120	164	47	3.10E-12
1R-MYB (MYB-Related)	*Lus.scaffold17.294*	403	445	45	3.50E-12
1R-MYB (MYB-Related)	*Lus.scaffold20.153*	121	165	47	3.10E-12
1R-MYB (MYB-Related)	*Lus.scaffold2.383*	5	56	52	4.40E-12
1R-MYB (MYB-Related)	*Lus.scaffold24.145*	86	130	47	3.40E-12
1R-MYB (MYB-Related)	*Lus.scaffold57.65*	86	130	47	3.90E-12
1R-MYB (MYB-Related)	*Lus.scaffold8.98*	102	146	47	4.80E-12
1R-MYB (MYB-Related)	*Lus.scaffold67.158*	105	149	47	5.30E-12
1R-MYB (MYB-Related)	*Lus.scaffold81.161*	76	120	47	7.20E-12
1R-MYB (MYB-Related)	*Lus.scaffold3.122*	5	55	51	1.10E-11
1R-MYB (MYB-Related)	*Lus.scaffold56.300*	249	287	43	1.20E-11
1R-MYB (MYB-Related)	*Lus.scaffold139.87*	363	406	46	1.70E-11
1R-MYB (MYB-Related)	*Lus.scaffold171.57*	241	292	51	4.80E-11
1R-MYB (MYB-Related)	*Lus.scaffold231.80*	95	139	45	1.40E-11
1R-MYB (MYB-Related)	*Lus.scaffold72.208*	97	141	45	1.50E-11
1R-MYB (MYB-Related)	*Lus.scaffold16.326*	132	172	44	3.40E-11
1R-MYB (MYB-Related)	*Lus.scaffold329.24*	242	293	51	6.80E-11
1R-MYB (MYB-Related)	*Lus.scaffold17.187*	238	289	51	2.80E-10
1R-MYB (MYB-Related)	*Lus.scaffold68.149*	59	103	45	1.80E-11
1R-MYB (MYB-Related)	*Lus.scaffold81.3*	252	290	43	2.40E-11
1R-MYB (MYB-Related)	*Lus.scaffold43.129*	59	103	45	3.00E-11
1R-MYB (MYB-Related)	*Lus.scaffold110.139*	61	105	45	3.20E-11
1R-MYB (MYB-Related)	*Lus.scaffold13.475*	161	205	45	2.70E-11
1R-MYB (MYB-Related)	*Lus.scaffold60.159*	403	445	45	4.00E-11
1R-MYB (MYB-Related)	*Lus.scaffold294.5*	15	65	51	1.00E-10
1R-MYB (MYB-Related)	*Lus.scaffold357.8*	15	65	51	1.00E-10
1R-MYB (MYB-Related)	*Lus.scaffold16.475*	327	374	50	9.10E-11
1R-MYB (MYB-Related)	*Lus.scaffold55.343*	18	54	37	2.60E-09
1R-MYB (MYB-Related)	*Lus.scaffold117.69*	14	64	51	3.40E-10
1R-MYB (MYB-Related)	*Lus.scaffold169.103*	14	64	51	3.40E-10
1R-MYB (MYB-Related)	*Lus.scaffold83.7*	568	617	50	2.30E-10
1R-MYB (MYB-Related)	*Lus.scaffold43.154*	31	71	43	1.10E-09
1R-MYB (MYB-Related)	*Lus.scaffold98.38*	258	309	51	3.30E-10
1R-MYB (MYB-Related)	*Lus.scaffold159.9*	62	104	45	2.50E-10
1R-MYB (MYB-Related)	*Lus.scaffold29.148*	119	154	44	1.60E-09
1R-MYB (MYB-Related)	*Lus.scaffold11.162*	216	267	51	4.40E-09
1R-MYB (MYB-Related)	*Lus.scaffold45.334*	216	266	50	7.80E-10
1R-MYB (MYB-Related)	*Lus.scaffold218.70*	33	81	50	5.90E-10
1R-MYB (MYB-Related)	*Lus.scaffold48.34*	142	191	50	5.60E-10
1R-MYB (MYB-Related)	*Lus.scaffold16.469*	51	93	44	7.20E-10
1R-MYB (MYB-Related)	*Lus.scaffold140.135*	126	174	50	7.10E-10
1R-MYB (MYB-Related)	*Lus.scaffold398.9*	107	155	50	5.90E-10
1R-MYB (MYB-Related)	*Lus.scaffold8.364*	216	266	50	7.70E-10
1R-MYB (MYB-Related)	*Lus.scaffold135.69*	106	154	50	5.90E-10
1R-MYB (MYB-Related)	*Lus.scaffold353.26*	49	91	44	7.30E-10
1R-MYB (MYB-Related)	*Lus.scaffold39.28*	43	75	38	2.30E-09
1R-MYB (MYB-Related)	*Lus.scaffold431.15*	181	230	50	6.80E-10
1R-MYB (MYB-Related)	*Lus.scaffold69.293*	167	216	50	6.30E-10
1R-MYB (MYB-Related)	*Lus.scaffold196.27*	126	174	50	7.40E-10
1R-MYB (MYB-Related)	*Lus.scaffold124.40*	248	298	51	9.40E-10
1R-MYB (MYB-Related)	*Lus.scaffold148.45*	5	56	52	1.30E-09
1R-MYB (MYB-Related)	*Lus.scaffold276.37*	33	81	50	1.00E-09
1R-MYB (MYB-Related)	*Lus.scaffold16.14*	67	117	51	1.30E-09
1R-MYB (MYB-Related)	*Lus.scaffold192.66*	78	128	51	1.50E-09
1R-MYB (MYB-Related)	*Lus.scaffold238.4*	5	57	52	1.60E-09
1R-MYB (MYB-Related)	*Lus.scaffold137.103*	16	66	52	2.40E-09
1R-MYB (MYB-Related)	*Lus.scaffold25.188*	468	517	50	1.80E-09
1R-MYB (MYB-Related)	*Lus.scaffold39.372*	252	294	49	6.70E-06
1R-MYB (MYB-Related)	*Lus.scaffold278.28*	27	77	51	1.70E-09
1R-MYB (MYB-Related)	*Lus.scaffold102.35*	47	96	51	2.10E-09
1R-MYB (MYB-Related)	*Lus.scaffold77.192*	47	96	51	2.10E-09

1R-MYB (MYB-Related)	*Lus.scaffold3.670*	166	215	51	2.90E-08
1R-MYB (MYB-Related)	*Lus.scaffold8.276*	46	78	38	1.20E-09
1R-MYB (MYB-Related)	*Lus.scaffold50.245*	55	106	51	1.00E-08
1R-MYB (MYB-Related)	*Lus.scaffold265.26*	292	341	50	2.70E-09
1R-MYB (MYB-Related)	*Lus.scaffold91.35*	38	88	51	2.10E-09
1R-MYB (MYB-Related)	*Lus.scaffold14.412*	5	55	51	4.80E-09
1R-MYB (MYB-Related)	*Lus.scaffold210.38*	28	79	52	4.40E-09
1R-MYB (MYB-Related)	*Lus.scaffold12.274*	5	55	51	5.70E-09
1R-MYB (MYB-Related)	*Lus.scaffold152.117*	24	76	54	5.50E-09
1R-MYB (MYB-Related)	*Lus.scaffold8.186*	309	358	50	4.70E-09
1R-MYB (MYB-Related)	*Lus.scaffold50.259*	46	78	38	5.20E-09
1R-MYB (MYB-Related)	*Lus.scaffold135.106*	26	76	51	6.70E-09
1R-MYB (MYB-Related)	*Lus.scaffold220.64*	22	64	45	6.20E-09
1R-MYB (MYB-Related)	*Lus.scaffold579.3*	22	64	45	6.20E-09
1R-MYB (MYB-Related)	*Lus.scaffold34.70*	199	249	50	1.00E-08
1R-MYB (MYB-Related)	*Lus.scaffold70.63*	200	250	50	1.00E-08
1R-MYB (MYB-Related)	*Lus.scaffold199.23*	393	441	50	9.70E-09
1R-MYB (MYB-Related)	*Lus.scaffold66.25*	54	104	51	9.60E-09
1R-MYB (MYB-Related)	*Lus.scaffold3.430*	468	517	50	1.20E-08
1R-MYB (MYB-Related)	*Lus.scaffold43.252*	17	67	51	1.10E-08
1R-MYB (MYB-Related)	*Lus.scaffold149.50*	55	105	51	9.60E-09
1R-MYB (MYB-Related)	*Lus.scaffold2.380*	308	357	50	1.20E-08
1R-MYB (MYB-Related)	*Lus.scaffold13.351*	104	153	50	1.90E-08
1R-MYB (MYB-Related)	*Lus.scaffold76.188*	4	54	51	1.50E-08
1R-MYB (MYB-Related)	*Lus.scaffold550.3*	4	54	51	1.50E-08
1R-MYB (MYB-Related)	*Lus.scaffold15.206*	4	54	51	1.70E-08
1R-MYB (MYB-Related)	*Lus.scaffold107.125*	49	100	51	2.70E-08
1R-MYB (MYB-Related)	*Lus.scaffold282.42*	275	326	51	2.50E-08
1R-MYB (MYB-Related)	*Lus.scaffold79.63*	148	198	50	2.10E-08
1R-MYB (MYB-Related)	*Lus.scaffold239.47*	82	132	51	3.00E-08
1R-MYB (MYB-Related)	*Lus.scaffold110.170*	33	71	44	3.70E-08
1R-MYB (MYB-Related)	*Lus.scaffold0.280*	197	247	50	2.40E-08
1R-MYB (MYB-Related)	*Lus.scaffold64.41*	175	226	51	3.80E-08
1R-MYB (MYB-Related)	*Lus.scaffold29.291*	5	57	52	3.90E-08
1R-MYB (MYB-Related)	*Lus.scaffold66.137*	275	326	51	3.50E-08
1R-MYB (MYB-Related)	*Lus.scaffold272.58*	119	161	44	1.50E-06
1R-MYB (MYB-Related)	*Lus.scaffold70.144*	224	271	50	5.40E-08
1R-MYB (MYB-Related)	*Lus.scaffold32.6*	293	340	49	4.20E-08
1R-MYB (MYB-Related)	*Lus.scaffold3.712*	254	305	51	5.20E-08
1R-MYB (MYB-Related)	*Lus.scaffold671.3*	254	305	51	5.20E-08
1R-MYB (MYB-Related)	*Lus.scaffold483.6*	87	137	53	5.20E-08
1R-MYB (MYB-Related)	*Lus.scaffold383.3*	96	144	51	6.10E-08
1R-MYB (MYB-Related)	*Lus.scaffold127.164*	91	139	51	6.50E-08
1R-MYB (MYB-Related)	*Lus.scaffold159.59*	439	481	45	1.20E-07
1R-MYB (MYB-Related)	*Lus.scaffold96.179*	87	137	53	6.40E-08
1R-MYB (MYB-Related)	*Lus.scaffold255.33*	479	522	44	8.40E-08
1R-MYB (MYB-Related)	*Lus.scaffold22.118*	166	213	49	1.10E-07
1R-MYB (MYB-Related)	*Lus.scaffold170.55*	13	58	49	7.00E-08
1R-MYB (MYB-Related)	*Lus.scaffold34.146*	224	271	49	1.20E-07
1R-MYB (MYB-Related)	*Lus.scaffold274.2*	13	58	49	7.20E-08
1R-MYB (MYB-Related)	*Lus.scaffold31.353*	275	326	51	1.10E-07
1R-MYB (MYB-Related)	*Lus.scaffold41.211*	176	225	51	1.10E-07
1R-MYB (MYB-Related)	*Lus.scaffold532.3*	472	520	50	1.10E-07
1R-MYB (MYB-Related)	*Lus.scaffold55.302*	472	520	50	1.10E-07
1R-MYB (MYB-Related)	*Lus.scaffold135.175*	714	764	53	1.30E-07
1R-MYB (MYB-Related)	*Lus.scaffold257.26*	20	71	51	1.30E-07
1R-MYB (MYB-Related)	*Lus.scaffold34.185*	4	28	25	1.60E-07
1R-MYB (MYB-Related)	*Lus.scaffold39.263*	14	41	31	1.60E-07
1R-MYB (MYB-Related)	*Lus.scaffold139.57*	277	328	53	1.30E-07
1R-MYB (MYB-Related)	*Lus.scaffold332.20*	240	291	51	1.60E-07
1R-MYB (MYB-Related)	*Lus.scaffold255.9*	13	58	49	1.50E-07

1R-MYB (MYB-Related)	*Lus.scaffold117.105*	670	698	39	9.20E-08
1R-MYB (MYB-Related)	*Lus.scaffold122.141*	13	58	49	1.50E-07
1R-MYB (MYB-Related)	*Lus.scaffold68.36*	209	258	50	2.30E-07
1R-MYB (MYB-Related)	*Lus.scaffold94.61*	225	275	50	2.20E-07
1R-MYB (MYB-Related)	*Lus.scaffold20.31*	498	546	50	2.10E-07
1R-MYB (MYB-Related)	*Lus.scaffold76.49*	620	668	50	2.60E-07
1R-MYB (MYB-Related)	*Lus.scaffold56.2*	259	310	51	3.00E-07
1R-MYB (MYB-Related)	*Lus.scaffold11.213*	130	181	51	3.70E-07
1R-MYB (MYB-Related)	*Lus.scaffold35.34*	128	179	51	4.50E-07
1R-MYB (MYB-Related)	*Lus.scaffold140.1*	42	92	51	4.00E-07
1R-MYB (MYB-Related)	*Lus.scaffold227.46*	234	281	48	7.60E-07
1R-MYB (MYB-Related)	*Lus.scaffold43.251*	44	94	51	4.10E-07
1R-MYB (MYB-Related)	*Lus.scaffold124.143*	1067	1114	50	5.30E-07
1R-MYB (MYB-Related)	*Lus.scaffold1.57*	221	270	51	4.40E-07
1R-MYB (MYB-Related)	*Lus.scaffold40.135*	226	274	50	5.20E-07
1R-MYB (MYB-Related)	*Lus.scaffold24.242*	20	71	51	5.60E-07
1R-MYB (MYB-Related)	*Lus.scaffold2.313*	238	289	51	5.40E-07
1R-MYB (MYB-Related)	*Lus.scaffold23.348*	222	271	51	5.00E-07
1R-MYB (MYB-Related)	*Lus.scaffold209.81*	38	88	52	5.20E-07
1R-MYB (MYB-Related)	*Lus.scaffold221.28*	173	222	51	5.40E-07
1R-MYB (MYB-Related)	*Lus.scaffold119.114*	258	309	51	2.90E-07
1R-MYB (MYB-Related)	*Lus.scaffold255.12*	11	55	47	4.40E-07
1R-MYB (MYB-Related)	*Lus.scaffold122.10*	562	611	50	7.80E-07
1R-MYB (MYB-Related)	*Lus.scaffold4.116*	573	622	50	8.00E-07
1R-MYB (MYB-Related)	*Lus.scaffold77.71*	89	133	54	8.70E-07
1R-MYB (MYB-Related)	*Lus.scaffold73.68*	192	243	51	1.00E-06
1R-MYB (MYB-Related)	*Lus.scaffold405.12*	102	152	51	7.80E-07
1R-MYB (MYB-Related)	*Lus.scaffold100.8*	192	243	51	1.10E-06
1R-MYB (MYB-Related)	*Lus.scaffold43.200*	305	361	60	9.90E-07
1R-MYB (MYB-Related)	*Lus.scaffold140.48*	301	357	60	1.20E-06
1R-MYB (MYB-Related)	*Lus.scaffold348.8*	1064	1111	50	1.30E-06
1R-MYB (MYB-Related)	*Lus.scaffold29.38*	41	88	50	2.20E-06
1R-MYB (MYB-Related)	*Lus.scaffold291.30*	585	634	50	1.70E-06
1R-MYB (MYB-Related)	*Lus.scaffold170.51*	12	55	46	1.50E-06
1R-MYB (MYB-Related)	*Lus.scaffold276.55*	526	575	50	2.30E-06
1R-MYB (MYB-Related)	*Lus.scaffold95.49*	242	293	51	2.20E-06
1R-MYB (MYB-Related)	*Lus.scaffold218.54*	527	576	50	2.20E-06
1R-MYB (MYB-Related)	*Lus.scaffold1.403*	13	59	50	3.40E-06
1R-MYB (MYB-Related)	*Lus.scaffold18.226*	60	100	44	3.00E-06
1R-MYB (MYB-Related)	*Lus.scaffold358.11*	129	171	44	4.00E-06
1R-MYB (MYB-Related)	*Lus.scaffold62.1*	1662	1715	56	6.00E-06
1R-MYB (MYB-Related)	*Lus.scaffold31.57*	236	284	49	4.00E-06
1R-MYB (MYB-Related)	*Lus.scaffold63.218*	276	327	51	3.30E-06
1R-MYB (MYB-Related)	*Lus.scaffold74.62*	13	59	50	3.50E-06
1R-MYB (MYB-Related)	*Lus.scaffold2.577*	562	611	50	5.00E-06
1R-MYB (MYB-Related)	*Lus.scaffold18.231*	60	106	48	4.70E-06
1R-MYB (MYB-Related)	*Lus.scaffold144.21*	558	607	50	4.90E-06
1R-MYB (MYB-Related)	*Lus.scaffold102.153*	624	668	49	5.10E-06
1R-MYB (MYB-Related)	*Lus.scaffold59.164*	129	171	44	9.40E-06
1R-MYB (MYB-Related)	*Lus.scaffold26.35*	58	104	49	7.70E-06
1R-MYB (MYB-Related)	*Lus.scaffold156.84*	19	62	47	9.10E-06

Note: This table describes the gene ID of each member of the flax MYB family and the relative position of each MYB domain in the amino acid sequence of the gene. Alignment start indicates the start position of the MYB domain predicted by HMMER software in the amino acid sequence, and alignment end indicates the end position. HMM length indicates the length of the MYB domain, and Conditional E-value indicates the value when the HMMER software matched the domain.

Supplementary Table S2 Statistical table of sequencing quality of flax varieties

Variety code	Sample	Error rate (%)	Q20 (%)	Q30 (%)	GC content (%)	Sample	Error rate (%)	Q20 (%)	Q30 (%)	GC content (%)
A	CK1_1	0.0258	97.69	93.49	48.18	SD1_1	0.0258	97.68	93.46	49.65
A	CK1_2	0.026	97.59	93.24	49.39	SD1_2	0.0258	97.65	93.48	48.86
A	CK1_3	0.0258	97.68	93.46	47.46	SD1_3	0.0261	97.57	93.26	48.7
B	CK2_1	0.0258	97.69	93.48	48.68	SD2_1	0.0257	97.72	93.55	49.37
B	CK2_2	0.0261	97.6	93.22	48.6	SD2_2	0.0253	97.87	93.95	49.39
B	CK2_3	0.0255	97.82	93.76	48.59	SD2_3	0.0255	97.82	93.74	49.44
C	CK3_1	0.0263	97.54	93.03	48.66	SD3_1	0.0256	97.81	93.66	49.22
C	CK3_2	0.0257	97.77	93.58	48.53	SD3_2	0.0258	97.72	93.48	48.67
C	CK3_3	0.0256	97.78	93.64	48.64	SD3_3	0.0254	97.88	93.95	48.94
D	CK4_1	0.0258	97.72	93.52	48.19	SD4_1	0.0257	97.74	93.55	48.51
D	CK4_2	0.0256	97.77	93.66	48.62	SD4_2	0.0254	97.91	93.83	48.65
D	CK4_3	0.0257	97.72	93.57	48.48	SD4_3	0.0259	97.67	93.42	48.78
E	CK5_1	0.0253	97.88	93.94	48.47	SD5_1	0.0254	97.85	93.84	48.53
E	CK5_2	0.0257	97.73	93.58	48.33	SD5_2	0.0259	97.69	93.42	48.93
E	CK5_3	0.0255	97.81	93.79	48.34	SD5_3	0.0256	97.78	93.65	48.96
F	CK6_1	0.0261	97.59	93.23	48.28	SD6_1	0.0255	97.8	93.73	48.26
F	CK6_2	0.0255	97.81	93.72	48.09	SD6_2	0.0255	97.81	93.74	48.28
F	CK6_3	0.0255	97.79	93.73	48.17	SD6_3	0.026	97.68	93.29	48.4
G	CK7_1	0.026	97.61	93.25	48.08	SD7_1	0.0257	97.73	93.56	47.99
G	CK7_2	0.0254	97.87	93.88	48.25	SD7_2	0.0256	97.78	93.67	48.54
G	CK7_3	0.0255	97.82	93.81	48.06	SD7_3	0.026	97.63	93.28	48.33
H	CK10_1	0.0254	97.85	93.87	48.15	SD10_1	0.0254	97.89	93.9	48.87
H	CK10_2	0.0257	97.75	93.63	47.61	SD10_2	0.0261	97.6	93.2	46.93
H	CK10_3	0.0255	97.78	93.75	48.08	SD10_3	0.0258	97.73	93.53	48.21
I	CK19_1	0.0258	97.7	93.56	48.08	SD19_1	0.0258	97.72	93.45	46.27
I	CK19_2	0.0257	97.73	93.57	47.52	SD19_2	0.0259	97.66	93.38	48.82
I	CK19_3	0.0257	97.71	93.54	48.2	SD19_3	0.0258	97.73	93.53	48.47
G	CK20_1	0.0251	97.98	94.11	48.57	SD20_1	0.0255	97.83	93.79	49.08
G	CK20_2	0.0258	97.71	93.5	47.31	SD20_2	0.0259	97.67	93.38	48.81
G	CK20_3	0.0257	97.72	93.51	47.49	SD20_3	0.0257	97.76	93.56	48.74
K	CK21_1	0.0256	97.76	93.65	47.55	SD21_1	0.0261	97.59	93.19	48.34
K	CK21_2	0.0255	97.79	93.73	47.74	SD21_2	0.0262	97.56	93.06	48.34
K	CK21_3	0.0255	97.8	93.76	47.79	SD21_3	0.0263	97.51	92.99	48.43
L	CK22_1	0.0261	97.58	93.21	48.11	SD22_1	0.0255	97.84	93.81	48.66
L	CK22_2	0.0252	97.93	94.06	48.28	SD22_2	0.0257	97.75	93.63	48.15
L	CK22_3	0.0255	97.8	93.79	48.35	SD22_3	0.0255	97.83	93.82	48.55

M	CK23_1	0.0256	97.78	93.71	48.62	SD23_1	0.0253	97.88	93.94	48.74
M	CK23_2	0.0258	97.7	93.48	48.66	SD23_2	0.0253	97.88	93.95	48.64
M	CK23_3	0.0254	97.82	93.84	48.54	SD23_3	0.0261	97.6	93.24	49.13
N	CK26_1	0.0255	97.8	93.73	48.48	SD26_1	0.026	97.63	93.33	48.64
N	CK26_2	0.0259	97.65	93.29	48.31	SD26_2	0.0255	97.83	93.76	48.55
N	CK26_3	0.0256	97.79	93.69	47.77	SD26_3	0.0261	97.57	93.18	48.66
O	CK27_1	0.0253	97.9	93.91	48.57	SD27_1	0.0264	97.48	92.94	48.22
O	CK27_2	0.0259	97.65	93.33	48.65	SD27_2	0.0257	97.73	93.56	48.77
O	CK27_3	0.0254	97.84	93.81	48.49	SD27_3	0.026	97.6	93.25	48.61
P	CK28_1	0.0256	97.79	93.69	48.21	SD28_1	0.0256	97.79	93.67	48.56
P	CK28_2	0.0261	97.57	93.17	48.2	SD28_2	0.0268	97.33	92.61	45.99
P	CK28_3	0.0258	97.7	93.52	48.74	SD28_3	0.0258	97.68	93.5	47.85
Q	CK29_1	0.0255	97.79	93.75	48.12	SD29_1	0.0263	97.51	93.05	47.96
Q	CK29_2	0.0259	97.67	93.36	48.42	SD29_2	0.0258	97.68	93.44	48.33
Q	CK29_3	0.0255	97.82	93.74	48.23	SD29_3	0.0256	97.77	93.65	47.93
R	CK30_1	0.0257	97.71	93.53	48.69	SD30_1	0.0252	97.93	94.01	48.32
R	CK30_2	0.0253	97.91	93.95	48.83	SD30_2	0.0265	97.44	92.83	48.55
R	CK30_3	0.0255	97.81	93.79	48.57	SD30_3	0.0258	97.69	93.42	48.1
S	CK31_1	0.0256	97.79	93.71	48.45	SD31_1	0.026	97.64	93.31	47.97
S	CK31_2	0.0259	97.67	93.39	48.54	SD31_2	0.0258	97.71	93.48	48.62
S	CK31_3	0.0253	97.93	93.97	48.59	SD31_3	0.0259	97.68	93.41	48.55
T	CK32_1	0.0254	97.83	93.87	48.71	SD32_1	0.0258	97.7	93.42	48.05
T	CK32_2	0.0255	97.8	93.78	47.66	SD32_2	0.0264	97.47	92.94	48.71
T	CK32_3	0.0258	97.69	93.5	47.79	SD32_3	0.0262	97.54	93.16	48.22

Note: CK means that this sample is the control group data of this variety, SD means that this sample is the drought group data of this variety, and each group of data of each variety is set with 3 replicates, denoted by _1, _2 and _3. Error rate (%): average error rate of sequencing bases corresponding to quality control data.

Supplementary Table S3 Annotated MYB gene statistics for each GO term

GO term	Gene ID	Variety code	Number of DEGs	Subfamily
biological regulation	*Lus.scaffold48.34*	A, B, D, E, K, M	6	1R-MYB
cellular component organization or biogenesis	*Lus.scaffold37.48*	B, E, K, M	4	2R-MYB
cellular process	*Lus.scaffold200.70*	A, B, E, K, M	5	1R-MYB
developmental process	*Lus.scaffold200.70*	A, B, E, K, M	5	1R-MYB
growth	*Lus.scaffold37.48*	B, E, K, M	4	2R-MYB
localization	*Lus.scaffold12.274*	B, M	2	1R-MYB
metabolic process	*Lus.scaffold440.7*	A, D, E, L, T	5	2R-MYB
reproduction	*Lus.scaffold29.148*	B	1	1R-MYB
reproductive process	*Lus.scaffold37.48*	B, E, K, M	4	2R-MYB
response to stimulus	*Lus.scaffold437.3*	A, B, K, T	4	1R-MYB
cell part	*Lus.scaffold80.38*	A, B, D, E, L, M, T	7	2R-MYB
extracellular region	*Lus.scaffold199.23*	B, E, M	3	1R-MYB
membrane	*Lus.scaffold39.372*	A, B, M	3	1R-MYB
membrane part	*Lus.scaffold39.372*	A, B, M	3	1R-MYB
organelle	*Lus.scaffold80.38*	A, B, D, E, L, M, T	7	2R-MYB
organelle part	*Lus.scaffold29.291*	E	1	1R-MYB
protein-containing complex	*Lus.scaffold29.291*	E	1	1R-MYB
binding	*Lus.scaffold35.141*	A, B, D, E, K, L, M	7	2R-MYB
catalytic activity	*Lus.scaffold440.7*	A, D, E, L, T	5	2R-MYB
transcription regulator activity	*Lus.scaffold48.34*	A, B, D, E, K, M	6	1R-MYB

Note: The table lists the MYB gene with the most frequent occurrence of each GO term in flax varieties. Column 3 represents the flax variety with the gene as DEGs, column 4 represents the number of flax varieties with the gene as DEGs, and column 5 represents the MYB subfamily to which the gene belongs.

Supplementary Table S4 Basic information of flax varieties

Number	Variety	Provenance	Type
1	Longya No. 8	Gansu, China	Drought resistant variety
2	Longya No.10	Gansu, China	Drought resistant variety
3	Dingya No.15	Gansu, China	Drought resistant variety
4	Dingxi No.17	Gansu, China	Drought resistant variety
5	Lixian	Gansu, China	Drought resistant variety
6	Gao Lan Bai	Gansu, China	Drought resistant variety
7	Zhangya No.1	Hebei, China	Drought resistant variety
8	Zhangya No.2	Hebei, China	Drought resistant variety
9	Baxuan No.3	Hebei, China	Drought resistant variety
10	Baya No.15	Hebei, China	Drought resistant variety
11	Ningya No.15	Ningxia, China	Drought resistant variety
12	Ningya No.17	Ningxia, China	Drought resistant variety
13	Ningya No.19	Ningxia, China	Drought resistant variety
14	Yiya No.4	Xinjiang, China	Drought resistant variety
15	Yiya No.5	Xinjiang, China	Drought resistant variety
16	Sha Chen Zao Shu Zhong Hong	Xinjiang, China	Drought resistant variety
17	R43	Inner Mongolia, China	Drought resistant variety
18	BGOLDXREDWING44X3	USA	Drought resistant variety
19	Neiya No.9	Inner Mongolia, China	Water sensitive variety
20	Longza No.1	Gansu, China	Water sensitive variety

Supplementary Table S5 Comparison results of ‘Strategy 4’ preference genes and *Arabidopsis thaliana* MYB family homologous genes using the Blastp algorithm

Query id	Subject id	% identity	Alignment length	Mismatches	Gap openings	Q. Start	Q. End	S. Start	S. End	E-value	Bit score
Lus.scaffold70.240	*AtMYB88*	57.173	481	175	12	24	490	21	484	1.82E-174	493
Lus.scaffold144.21	*At-TBP-TRFL9 (AT3G12560.1)*	45.401	674	267	21	1	648	1	599	1.58E-155	457
Lus.scaffold381.22	*AT4G34430.3*	45.221	973	412	25	1	940	58	942	0	657
Lus.scaffold4.116	*At-TBP-TRFL2 (AT1G07540.1)*	48.471	425	179	16	245	661	224	616	1.29E-98	310
Lus.scaffold8.186	*AT4G18020.4*	44.948	485	197	16	1	458	1	442	7.07E-116	346

Note: The list shows the *Arabidopsis thaliana* genes with the highest Blast alignment value for each preference gene in ‘Strategy 4’.

Supplementary Table S6 Primer sequences used in the q-PCR experiment

Gene Name	Primer sequences
Actin-F	TCCAGGCCGTTCTTTCTCTA
Actin-R	CTGTAAGGTCACGACCAGCA
Lus.scaffold70.240-F	AGGAGATGAGGTCACAAGT
Lus.scaffold70.240-R	GGTGCTGAGTATGATATGAGA
